# Dietary Patterns and New-Onset Diabetes Mellitus in Southwest China: A Prospective Cohort Study in the China Multi-Ethnic Cohort (CMEC)

**DOI:** 10.3390/nu16111636

**Published:** 2024-05-27

**Authors:** Yanqi Hu, Xianbin Ding, Liling Chen, Youxing Luo, Xin Liu, Xiaojun Tang

**Affiliations:** 1 School of Public Health, Research Center for Medical and Social Development, Chongqing Medical University, Chongqing 400016, China; 2022110579@stu.cqmu.edu.cn (Y.H.); 2022120818@stu.cqmu.edu.cn (Y.L.); 2021120782@stu.cqmu.edu.cn (X.L.); 2 Institute of Chronic Non-Communicable Disease Control and Prevention, Chongqing Center for Disease Control and Prevention, Chongqing 400042, China; xianbinding@126.com (X.D.); mbcllgz@163.com (L.C.)

**Keywords:** dietary pattern, food frequency questionnaire, Asia, diabetes

## Abstract

(1) Background: There is little known about the relationship between Dietary Approaches to Stop Hypertension (DASH) pattern and diabetes in cohort studies, and the dietary patterns in the Chongqing natural population are unknown. (2) Methods: 14,176 Chinese adults, aged 30–79 years old, participated in this prospective study, from September 2018 to October 2023. A dietary assessment was conducted using a food frequency questionnaire, and three main dietary patterns were extracted from the principal component analysis. DASH patterns were calculated by standards. (3) Results: During the 4.64 y follow-up, 875 developed diabetes (11.3/1000 person-years). Each posteriori diet pattern is named after its main dietary characteristics (meat pattern, dairy products–eggs pattern, and alcohol–wheat products pattern). The high consumption of DASH pattern diet reduced the risk of diabetes (Q5 vs. Q1 HR: 0.71; 95% CI: 0.40–0.56) while high consumption of alcohol–wheat product pattern diet was associated with a high risk of diabetes (Q5 vs. Q1 HR: 1.32; 95% CI: 1.04, 1.66). The other two dietary patterns were not associated with diabetes. In subgroup analysis, there was an interaction between DASH pattern and sex (P for interaction < 0.006), with a strong association in females. (4) Conclusions: DASH pattern may be associated with a reduced new-onset diabetes risk and Alcohol-wheat products pattern may be positively associated with new-onset diabetes. These findings may provide evidence for making dietary guidelines in southwest China to prevent diabetes.

## 1. Introduction

Diabetes mellitus (DM) is a common metabolic disease characterized by abnormal hyperglycemia. The latest report (2021) released by the International Diabetes Federation (IDF) estimated that 537 million (10.5%) adults aged 20–79 years old have DM around the world and that there is 1 diabetic in every 10 people [1]. The overall prevalence of diabetes in mainland China increased from 10.9% in 2013 to 12.8% in 2017 [2]. With the increasing prevalence of this chronic disease, the prevention of DM is urgently needed.

Individual food consumption as a lifestyle intervention can reduce the potential risks of diabetes (such as obesity, hyperlipemia, and abnormal serum glucose) by adjusting single dietary intake [3,4,5]. However, the effect of different combinatorial foods on the body may conflict with single food results. In recent years, there has been an increasing amount of research on dietary patterns. Analyses of dietary patterns can be approximately divided into a priori eating pattern studies and a posteriori eating pattern studies. Dietary approaches to stop hypertension (DASH), a well-known priori eating pattern, has been widely shown to be beneficial in reducing cardiovascular disease, especially in hypertension [6]. However, its research evidence regarding diabetes is scarce [7]. Unlike a priori eating patterns with established standards, the a posteriori pattern is structured based on regional data and multivariate statistical analysis tools [8]. Due to different socio-demographic characters in different regions, there are many studies on posteriori dietary patterns. In fact, it is now generally accepted that there is not a “one-size-fits-all” eating pattern for individuals with diabetes, and the American Diabetes Association (ADA) recommends that meal planning should be individualized [9].

The China Multi-Ethnic Cohort (CMEC) Study is a large-scale epidemiological study undertaken in Southwest China [10], while the study of the dietary pattern and diabetes was limited in cross-sectional observation. As one of the main areas in CMEC, Chongqing has a great deal of mountainous and hilly terrain, and it is one of the most humid areas in China. As well, the cuisine of Chongqing is characterized by spicy food. Therefore, the food pattern in this region is also different from that of southwestern China as a whole. Hence, this study aimed to establish food patterns and examine the association between dietary patterns and DM incidence in Chongqing, China.

## 2. Materials and Methods

### 2.1. Study Design and Study Population

**Figure 1:**
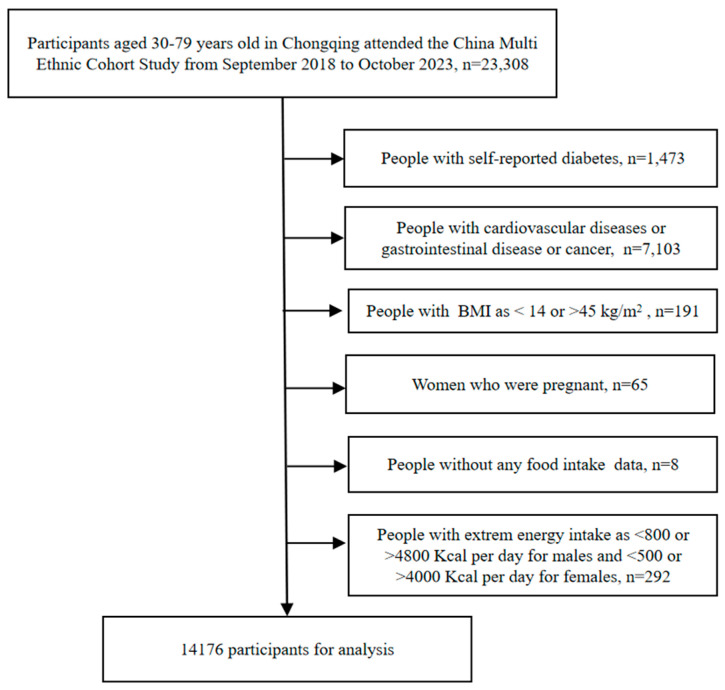
Flow Chart.

This prospective cohort study was conducted from September 2018 to October 2023 in Chongqing, a municipality in southwestern China. After multi-stage, stratified, community-based cluster sampling from 13 main districts and counties (districts and counties of the same grade), 23,308 Chinese adults of Han nationality, aged 30–79 years old, who had lived in the local area for half a year or more participated. Ethical approval was obtained from the Medical Ethics Review Committee of Sichuan University (K2016038) on 9 November 2016. All participants gave written informed consent. In the present study, a total of 14,176 potential participants were screened for eligibility (Figure 1) and people with the following conditions were excluded at baseline: (1) those with self-reported diabetes; (2) those who had cardiovascular diseases or gastrointestinal disease [11,12] or cancer; (3) those who have extreme value of BMI (<14 or >45 kg/m^2^); (4) those who were pregnant; (5) those without any food intake information; and (6) those with extreme energy intake (defined as <800 or >4800 kcal per day for males and <500 or >4000 kcal per day for females [13]).

### 2.2. Dietary Patterns Assessment

The baseline survey from September 2018 to January 2019 consisted of a tablet-based electronic questionnaire via face-to-face interviews, anthropometric measurements, and blood tests. More details regarding the questionnaire design are provided in Table S1. The Food Frequency Questionnaire (FFQ) mainly contains 13 crude common food items, including the quantity (average grams per meal according to standard serving size molds) and frequency (four frequency categories ranging from how many times per day to year) that they consumed during the past 12 months. In other sections, information regarding alcohol, tea, and beverages was collected by questions about current status, duration, types, and frequency. Cooking oil and salt were roughly recorded by asking about household consumption. In 2020, we conducted repeated FFQ and 24-h dietary recalls (24HDRs) to assess the reproducibility and validity of the baseline FFQ. The Interclass Correlation Coefficient (ICC) for reproducibility ranged from 0.25 (rice) to 0.68 (tea). The Spearman coefficients for validity ranged from 0.41 (fresh vegetables) to 0.63 (dairy products). More details can be seen in Table S4.

**Figure 2:**
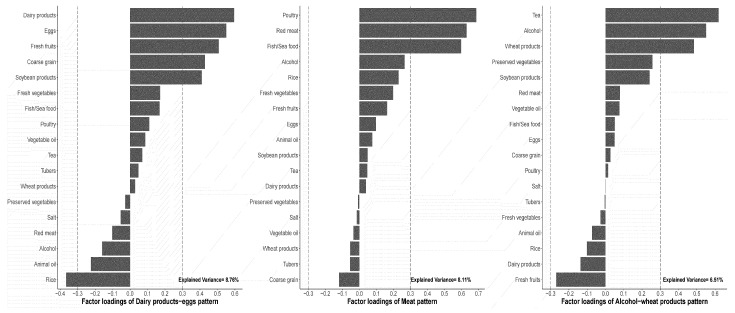
Factor loading matrix of dietary patterns by 18 food groups derived from a principal analysis from 14,716 participants from the baseline survey of the CME cohort study.

In the a posteriori dietary pattern, we first calculated the personal daily food intake (g/day) for 18 food categories, and then standardized the values by Z score. The Kaiser–Meyer–Olkin measure of sampling adequacy was 0.610 > 0.60, and Bartlett’s test of sphericity was significant (chi-square = 10,138.57, *p* < 0.001). Principal component analysis (PCA) was used to construct the a posteriori dietary patterns. After varimax rotation, we picked out three dietary patterns which met the statistical criteria of initial eigenvalue > 1 and food categories with factor loading coefficient > |3.0| as the patterns’ principal component (Figure 2). In the a priori dietary pattern, we focused on the DASH pattern and use a slightly adjusted calculation criteria to form the pattern (more information can be seen in Table S5). All the patterns were divided into quintiles for analysis.

### 2.3. Assessment of Covariates

Each participant enrolled in the CMEC study in the baseline was asked about socio-demographic characteristics (sex, age at recruitment, education level, marital status, occupation, household income), personal and family history of major diseases (cancer, CVD, diabetes, hypertension, etc.), lifestyle (smoking status, drinking status, tea consumption, dietary habits, physical activity), sleeping, and mental health using a standard questionnaire. Physical examinations were measured by medical professionals. The examination indicators include height, weight, waist circumference, gynecological B-ultrasound (uterus, ovary), and so on. Measurements of height and weight were taken using a vertical altimeter and an electronic scale without wearing shoes, hats, or heavy coats (accurate to 0.1 cm and 0.1 kg, respectively). Blood pressure was measured at a calm state (sit still for at least 5 min). Three consecutive sets of data were collected by an electronic sphygmomanometer, with an interval of 1 min. Intravenous blood samples were taken between 7 a.m. and 9:30 a.m. (fasting for at least 8 h). Then specimens were collected and transported to a third-party laboratory in dry ice packaging. Blood samples were tested by the laboratory, including fasting blood glucose (FBG), glycated hemoglobin (HbA1c), total cholesterol (TC), triglyceride (TG), Low density lipoprotein cholesterol (LDL-C), high-density lipoprotein cholesterol (HDL-C), etc.

### 2.4. Continuous Variables Converted to Categorical Variables

Except for the nutrients (daily energy, protein, fat and carbohydrate calculated by daily food categories intake; see the standard in Table S3), other variables were all converted to categorical variables. Age was divided into two groups: the youngsters (<60 years old) and the elderly (≥60 years old). In consideration of the few participants in the underweight or obese categories (*n* = 247 (1.7%) and *n* = 634 (4.5%), respectively) defined by BMI [14], we divided BMI into two groups: standard and below (<24 Kg/m^2^) and overweight and above (≥24 Kg/m^2^). Educational level was divided into three levels: primary school or below, middle school or high school, and high school or above. Weekly physical activity was categorized according to the Physical Activity Guidelines for Americans (2018) [15]: <12.0 METs-h per week as insufficient physical activity, ≥12.0 METs-h per week as sufficient physical activity. Frequency of eating spicy food was divided into three levels: <1 day per week as low-rate eating, 1–5 days per week as medium-rate eating, ≥6 days per week as high-rate eating. Smoking, alcohol, and tea were recognized as binary categorical variables (e.g., current smokers or not). According to the guidelines for the prevention and treatment of type 2 diabetes mellitus in China (2020) [16], five conventional biochemical indexes were divided into two categories. serum creatinine (Cr) was divided into two groups: 57–97 µmol/L among 20–59 years old and 57–111 µmol/L among 60–79 years old as normal in males, 41–73 µmol/L among 20–59 years old and 41–81 µmol/L among 60–79 years old as normal in females; beyond this range is abnormal. Systolic blood pressure (SBP) was divided into two groups: 90–140 mmHg as normal, <90 or >140 mmHg as abnormal. Diastolic blood pressure (DBP) was divided into two groups: 60–90 mmHg as normal, <60 or >90 mmHg as abnormal. Triglyceride (TG) was divided into two groups: 0.56~1.70 mmol/L as normal, <0.56 or >1.70 mmol/L as abnormal. Total cholesterol (TC) was divided into two groups: 2.84~5.68 mmol/L as normal, <2.84 or >5.68 mmol/L as abnormal. Low-density lipoprotein cholesterol (LDL-C) was divided into two groups: 2.10~3.10 mmol/L as normal, <2.10 or >3.10 as abnormal. High-density lipoprotein cholesterol (HDL-C) was divided into two groups: 1.14~1.76 mmol/L as normal, <1.14 or >1.76 mmol/L as abnormal.

### 2.5. Outcome Ascertainment

The outcomes in this study were collected at two follow-up visits in 2021 and 2023. Typical diabetic symptoms with fasting plasma glucose (FPG) ≥ 126 mg/dL (7.0 mmol/L) or 2-h plasma glucose (2-h PG) ≥ 200 mg/dL (11.1 mmol/L) or during oral glucose tolerance test (OGTT) or a random plasma glucose ≥ 200 mg/dL (11.1 mmol/L) or HbA1c (glycosylated hemoglobin) ≥ 6.3% were the criteria for diagnosing diabetes [16]. Participants with a doctor’s diagnosis information were considered new-onset diabetics. Monitoring systems or database formed in the routine work were used to supplement or confirm their diagnosis information.

### 2.6. Statistical Analyses

We used PCA to establish three dietary patterns and quintile the patterns and DASH pattern. The socio-demographic baseline characteristics, lifestyle factors, and family history of diabetes were compared across the quintiles of the dietary pattern scores, described by percentage (%) for categorical variables and means ± standard deviations (SDs) for continuous variables, by Student’s *t*-tests, Mann–Whitney U tests or Chi square tests as appropriate. COX regression analysis was used to calculate the hazard ratios (HRs) and 95% confidence interval (CI) for the risk of diabetes incidence across the quintiles of the dietary pattern scores with the lowest quintile as the reference category. A trend test was performed by containing median values for each quintile in the corresponding model. Model 1 adjusted for sex and age (<60 or ≥60 years old). Model 2 adjusted for region (urban or rural), educational level (primary school or below, Middle school or high school, high school above), household annual income (<20,000, 20,000–99,999, 10,000–19,999, ≥20,000 CNY/year), family history of diabetes, BMI (standard and below, overweight and above) based on Model 1. Model 3 adjusted for weekly physical activity (<12.0, ≥12.0 METs-h per week), smoking, drinking alcohol, drinking tea, eating spicy food (<1, 1–5, ≥6 days per week), daily total energy intake (kcal/day) based on Model 2. Model 4 adjusted for serum creatinine (SCr) (male in normal: 20–59 years old is 57–97 µmol/L, 60–79 years old is 57–111 µmol/L; female in normal: 20–59 years old is 41–73 µmol/L, 60–79 years old is 41–81 µmol/L, beyond this range is abnormal), systolic blood pressure (SBP) (90–140 mmHg for normal, beyond this range is abnormal), diastolic blood pressure (DBP) (60–90 mmHg for normal, beyond this range is abnormal), triglyceride (TG) (0.56~1.70 mmol/L for normal, beyond this range is abnormal), total cholesterol (TC) (2.84~5.68 mmol/L for normal, beyond this range is abnormal ), low-density lipoprotein cholesterol (LDL-C) (2.10~3.10 mmol/L for normal, beyond this range is abnormal), high-density lipoprotein cholesterol (HDL-C) (1.14~1.76 mmol/L for normal, beyond this range is abnormal) based on Model 3. We also used a stratified analysis to examine the relationship between various dietary patterns and the incidence of diabetes in 15 subgroups (sex, age, BMI, family history of diabetes, region, smoking status, alcohol status, the frequency of spicy food per week, SCR, SBP, DBP, TC, TGs, LDL-C, and HDL-C).

All the data were preprocessed in Excel software 2021 (edition 2404 Build 16.0.17531.20152)and imported to R version 4.2.3 for statistical analysis and plotting. The significance level was set at a *p*-value of <0.05 for two-sided testing. For the multiplicative interaction tests in the stratified analyses, the statistical significance was defined as *p* < 0.006.

## 3. Results

### 3.1. Baseline Description of Each Patterns

In Figure 2, three dietary patterns were extracted from the PCA method, explaining 8.76%, 8.11%, and 6.51% of the variation of food variable. The first pattern, which we named “dairy products–egg pattern”, was mainly characterized by a high intake of dairy products, eggs, fresh fruits, coarse grains, and soybean products and a low intake of rice. The second pattern, which we named the “meat pattern”, was mainly characterized by a high intake of poultry, red meat and its processed products, and fish/seafood. The third pattern, which we named the “alcohol–wheat product pattern”, was mainly characterized by a high intake of tea, alcohol, and wheat products. The slightly-adjusted DASH pattern was characterized by a high intake of vegetables, fruits, legumes, whole grains and dairy products and a low intake of red meat and its products and sodium (see Table S5 for establishment rules). A slightly adjusted DASH pattern was formed by the standard (Table S5). The patterns’ food intake distribution can be found in Figure S1.

Of the 14,173 participants, 875 developed diabetes (incidence rate: 11.3/1000 person-years) over a 4.64-y follow-up on average. At baseline, the mean age of the study subjects was 48.6 years old, and 54.5% of the subjects were women. In Table 1, compared with the participants in the lowest quintile of the Meat pattern, those with higher dietary pattern scores were more likely to be females, youngsters, current smokers, current drinkers, from rural, with a higher education and a higher household income; to engage in sufficient weekly physical activity; to have an overweight figure; and to regularly eat spicy food. Participants with higher meat pattern scores were more likely to have abnormal levels of TC, LDL-C, and DBP (*p* < 0.01). Participants with higher scores in the dairy products–egg pattern were more likely to be females and younger, to live in urban areas, to have a higher education attainment and a higher household income, to have a family history of diabetes, and to prefer smoking and drinking tea; these participants were thinner and drank alcohol and ate spicy food less. There were less abnormal levels of SBP, DBP, TC, and LDL-C among these people (*p* < 0.001). Elderly men who were more likely to smoke, drink tea and alcohol, and eat spicy food and had a family history of diabetes with a lower educational level and a insufficient weekly physical activity and lower BMI are more likely to be observed in the alcohol–wheat products pattern. As well, people with higher alcohol–wheat products pattern scores intended to have abnormal levels of SBP, DBP, TC, TG and LDL-C (*p* < 0.05). In the DASH pattern, younger and thinner women with a higher education attainment and a higher household income in urban region, who drink alcohol but did not smoke were the characteristics of the participants with a higher score. They were more likely to have normal levels of SBP, DBP, TC, TG, and LDL-C (*p* < 0.01).

### 3.2. Risk Analysis

In the univariate model (Table 2), a higher score of alcohol–wheat products pattern was significantly associated with an increased risk of incident diabetes in the sex + age adjusted models (Q5 vs. Q1 HR = 1.41; 95% CI: 1.14–1.75), the socio-demographic-adjusted model (Q5 vs. Q1 HR = 1.43; 95% CI: 1.51–1.77), the lifestyle-adjusted model (Q5 vs. Q1 HR = 1.34; 95% CI: 1.06–1.58), and the fully adjusted model (Q5 vs. Q1 HR = 1.32; 95% CI: 1.04–1.66). The highest quartile of the DASH pattern score showed a significantly lower risk of incident diabetes (Model 1: HR: 0.67; 95% CI: 0.54, 0.84; Model 2: HR: 0.71; 95% CI: 0.56, 0.90; Model 3: HR: 0.72; 95% CI: 0.57, 0.91; Model 4: HR: 0.71; 95% CI: 0.40, 0.56) compared with the lowest quartile of the dietary pattern score. However, we did not observe any significant association between meat pattern/dairy products–egg pattern and diabetes incidence in the full adjusted model (all *p*-value > 0.05).

### 3.3. Subgroup Analysis

**Figure 3:**
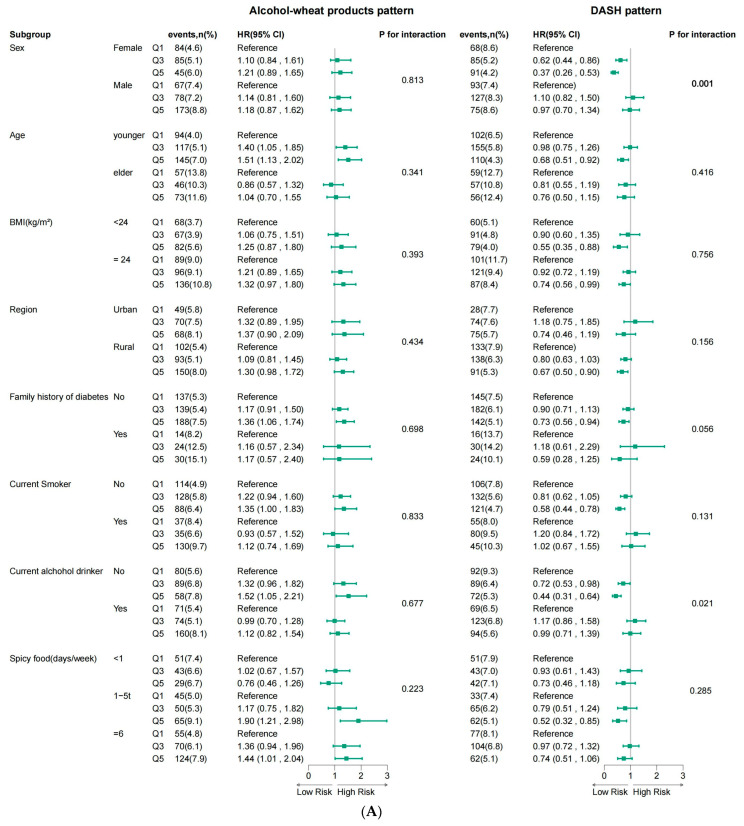

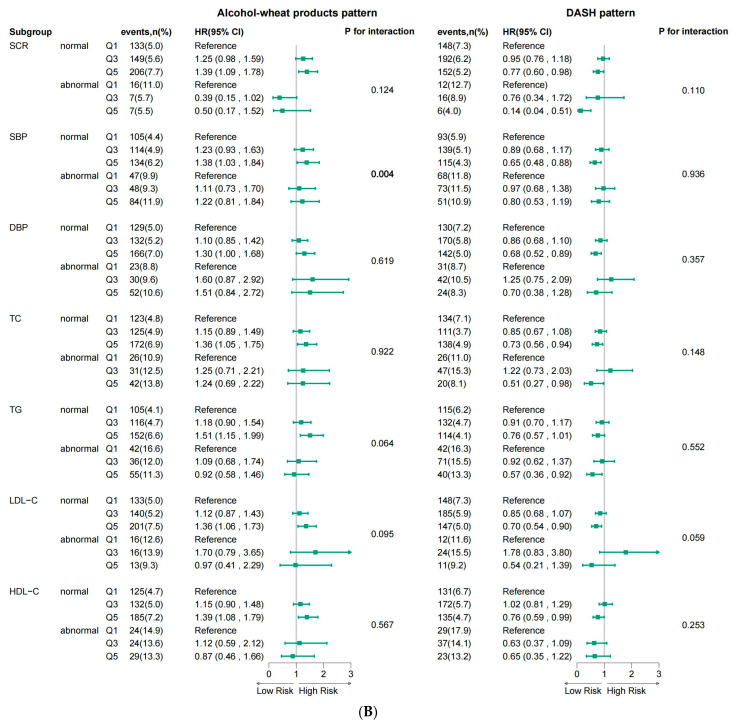
Forest map for the association between patterns and diabetes stratified by the age, sex, BMI, smoking, alcohol, spicy food (**A**) and SCR, SBP, DBP, TC, TG, LDL-C, and HDL-C (**B**).

We performed a further subgroup analysis of Model 4, which can be seen in a forest plot (Figure 3); we excluded subgroups of some variations that had no significant effect on diabetes. There was a statistical interaction between DASH and the sex of diabetes incidence (sex = female, Q5 vs. Q1: HR = 0.37; 95% CI: 0.26–0.53; p for interaction < 0.006). As for age, the DASH pattern had a lower risk of diabetes in youngsters (Q5 vs. Q1: HR = 0.68; 95% CI: 0.51–0.92), while in the alcohol–wheat product pattern, we observed a higher risk of diabetes (Q5 vs. Q1: HR = 1.51; 95% CI: 1.13–2.02). No matter how the BMI is, the highest score of DASH pattern was significantly associated with a low risk of incident diabetes (BMI < 24 kg/m^2^, Q5 vs. Q1: HR = 0.55; 95% CI: 0.35–0.88; BMI ≥ 24 kg/m^2^, Q5 vs. Q1: HR = 0.74; 95% CI: 0.56–0.99). However, the meat pattern still had no associations with diabetes, and the dairy products–egg pattern only in the sex and region subgroups had little effect on lowering risk of diabetes (sex = female, Q5 vs. Q1: HR = 0.58; 95% CI: 0.40–0.83; region = rural, Q5 vs. Q1: HR = 0.74; 95% CI: 0.55–0.99). More details can be seen in Table S6.

## 4. Discussion

In this prospective cohort study of adults from southwestern China, we identified three main dietary patterns by PCA and assessed the adherence to slight-adjusted DASH pattern. The alcohol–wheat product pattern—characterized by a high intake of tea, alcohol, and wheat products—had a positive association with incident diabetes. Both the dairy products–egg pattern, which was characterized by a high intake of dairy products, eggs, fresh fruits, coarse grain and soybean products and a low intake of rice, and the meat pattern, characterized by a high intake of poultry, red meat and its processed products, and fish/seafood, were not significantly associated with diabetes risk. There was a negative association between the diabetes events and the DASH pattern rich in vegetables, fruits, legumes, whole grains and dairy products and low in red meat and its products and sodium.

For the DASH pattern, almost all studies have shown its effective role in reducing cardiovascular outcomes [17,18]. However, little evidence of prospective studies is found in diabetes. CMEC has published an article related to this pattern in a cross-sectional study [19], which estimated that a slightly adjusted DASH pattern was a superior dietary recommendation to reduce cardiometabolic risks. We further confirmed this view for diabetes in this cohort under rigorous inclusion and exclusion criteria. A meta-analysis of six large prospective studies in 2013 established an inverse association between adherence to a DASH-like diet (rich in fruits, vegetables, and low-fat dairy products and low in SFA, total fat, cholesterol, refined grains, sweets, red meat, and salt) and risk of T2DM incidence (overall risk ratio (RR): 0.81; 95% CI: 0.72, 0.92) [20]. Similar results (RR: 0.80; 95% CI: 0.74, 0.86) were found in the most recent meta-analysis of 8 prospective studies (DASH is composed of 10 food groups: total grains or high-fiber grains, vegetables, fruits, total dairy or low-fat dairy, nuts, seeds, and legumes, low intake of meat, fats or oil, sweets) [21]. Recently, a national prospective cohort study in the USA has proved the DASH pattern (high intake of fruits, vegetables, nuts and legumes, low-fat dairy products, and whole grains and low intake of sodium, sweetened beverages, and red and processed meats) was statistically significantly associated with increased risk of type 2 diabetes (Q1 vs. Q5) [22], which was similar to our findings. A clinical trial shows adults with overweight or obesity, hypertension, and prediabetes or type 2 diabetes had improvements in SBP, glycemic control, and weight over a 4-month period combined with DASH pattern, which means the potential in preventing diabetes [23]. The evidence from RCTs examining the effect of the DASH diet on glycemic control is limited to GDM [7]. In subgroup analysis, we found that the prevention of diabetes was most significant in women (Q5 vs. Q1: HR: 0.37; 95% CI: 0.26, 0.53) and there’s an interaction between the sexes (*p* < 0.006). This may be one of the reasons that DASH diet studies found more in GDM. Moreover, we also observed the factor of drinking alcohol status had a slight effect on the interaction between the DASH pattern and new-onset diabetes (*p* < 0.05). In the ordinary DASH pattern in the clinical trial [24,25], alcohol consumption is limited for the rise of blood pressure. However, the alcohol consumption in the study of diabetes and DASH pattern is still unclear. Further study should be taken to analyze the relationship between alcohol consumption and the DASH pattern.

The alcohol–wheat product pattern is a unique diet pattern; we did not find any similar patterns in other studies. A cross-sectional study on the southeast coast of China finds a high-alcohol diet pattern characterized by high intake of alcohol, rice, wheat products, and eggs using K-means clustering analysis on the basis of the percent contribution to the total energy intake was not associated with type 2 diabetes (adjusted odds ratio (OR): 1.5; 95%CI: 1.0, 2.4) [26]. A male-specific diet pattern, characterized by a high intake of wheat products, coffee, juice, and fried dough foods and a low intake of fruits, poultry, fresh-water fish, and vegetables is associated with the risk of poor glycemic control in Chinese diabetic adults (Q4 vs. Q1, OR: 2.69; 95% CI: 1.76, 4.10) [13]. However, we did not find any statistical significance in the sex subgroup. It seems that we should pay attention to the particular intake on dietary patterns to explain the results. Clinical trials have confirmed that tea is effective intervention in diabetes [27]. And in the Alcohol-wheat products pattern, the tea intake in Q5 represents for one cup a day (3.77 g/day) which meant in a moderate tea drinking range. For alcohol, the relationship between alcohol and the development of type 2 diabetes follows a U-shaped relationship [13,28]. Regarding the lack of accepted limits to moderate drinking, large, prospective, epidemiological studies have shown that consumption rates of 5–20 g of alcohol a day are associated with a reduced risk of developing diabetes [27,29,30,31,32]. In our study, only alcohol intake of Q5 belongs to moderate level (mean: 16.79 g/day). In view of the grams of the daily intake of tea and alcohol, maybe this is the reason why the HR in Q5 is lower than that in Q4 (Q4 vs. Q1: adjusted HR in Model 4: 1.50; 95% CI: 1.20–1.86; Q5 vs. Q1: adjusted HR in Model 4: 1.32, 95% CI: 1.04–1.66). Moreover, as we all know, fresh fruits are beneficial for prevention of DM [33] and wheat products as a high-glycemic index (GI) food is positively correlated with insulin resistance, which is one of the important reasons for the development of diabetes [34]. In the alcohol–wheat product pattern, a low intake of fresh fruits was 93 g/day and a high intake of wheat products was 115.32 g/day in Q5 for average. As well, a high intake of preserved vegetables full of salt and soybean products were observed in this pattern. With an eye to the people characterized as elders tending to smoke and a lower educational degree and a insufficient weekly physical activity, this dietary pattern is a risk factor for diabetes without adequate nutrient intake.

When comes to the dairy products–egg pattern, significance was only found in the subgroup analysis of female and rural region (Table S6). The studies about dairy products are contradictory and highly controversial. Moreover, few studies report the relationship between eggs and diabetes. One cohort study in Iran discovered no relationship between a healthy diet pattern derived from PCA (with a higher load of whole grains, vegetables, and dairy products) and regression or progression from pre-DM [35]. Another study in Spain found no association between eggs and dairy patterns (characterized by high intake of eggs, dairy products, fats and red meat and low intake of salty snacks and soup) and diabetes-related metabolic markers in women [36]. We assumed that it may be the moderate intake for eggs and dairy products (49.17 g/day and 173.21 g/day) which result in moderate protein intake that does not cause a rise in blood glucose, though the overall combination seemed healthy, lead to the unobvious results. Our results on the meat pattern were consistent with similar meat patterns from some cohort studies in Asia (high intakes of fish, poultry, or red meat and its processed products and other staples as well as fresh fruit and vegetables) [37,38,39]. Nevertheless, there are still some studies different from us. A 14-y follow-up multiethnic study in the United States reported that a fat-and-meat dietary pattern was significantly associated with incident diabetes [40]. It is widely believed that red meat and its processed products is positively correlated with the incidence of T2DM [4]. Thus, the meat pattern should be studied in greater detail and depth in the future.

There are several limitations to our study. Firstly, our food classifications are crude and incomplete. Some food items related to diabetes such as nuts are not included in the FFQ. However, we will supplement this information in future studies. Secondly, we did not assess the effects of cooking styles and other tastes on eating patterns, which may lead to bias in results. In addition, because the PCA method depends on available data, the ability to reproduce the results in other study populations is limited. Not only that, we were not able to distinguish between type 1 and type 2 diabetes. Given the age of our subjects at baseline, the diabetes observed were more likely to be type 2 diabetes. There may have been measurement errors in the estimation of daily intake values, which may have resulted in weaker associations. Last but not the least, dietary investigations were not performed on every follow-up survey, so changes in dietary intake were not considered in this study. However, our study has some strengths. First of all, this is the first prospective cohort study in CMEC to explore the relationship between dietary patterns and diabetes which adds robust evidence to the results of previous cross-sectional study [19]. Then, we excluded people with any diseases that could cause reverse causation and provided stronger evidence for causality compared with other cohort designs. Moreover, we not only established the posteriori dietary patterns but also concluded the DASH pattern, which was seldom found in other studies in Asia. Finally, because of the Chongqing’s local cuisine is characterized by its spiciness, our study took into account of the frequency of spicy food as one of the important covariates, which was newly different from other studies on food patterns.

## 5. Conclusions

In conclusion, our study identified three dietary patterns by the PCA method and the established slightly adjusted DASH pattern. Among them, the DASH pattern showed the inverse correlation with diabetes, while the alcohol–wheat product pattern was positively associated with diabetes. The dairy products–egg pattern and meat pattern had no relationship with diabetes. Therefore, the DASH pattern may provide a solution for dietary guidance in diabetes prevention.

## Figures and Tables

**Table 1:** Socio-demographic and lifestyle characteristics by quintile category (Q1 and Q5) of the PCA-derived meat, dairy products–eggs, and alcohol–wheat products dietary pattern scores and DASH pattern scores and their respective simplified dietary pattern scores in the CME cohort study.

	Overall	Meat Pattern		^†^ *p*-Value	Dairy Products-Eggs Pattern		^†^ *p*-Value	Alcohol-Wheat Products Pattern		^†^ *p*-Value	DASH Pattern		^†^ *p*-Value
		Q1	Q5		Q1	Q5		Q1	Q5		Q1	Q5	
* Pattern score		−0.95 ± 0.25	1.48 ± 1.19	<0.001	−1.20 ± 0.35	1.50 ± 0.77	<0.001	−0.91 ± 0.32	1.43 ± 1.30	<0.001	13.22 ± 1.69	26.89 ± 1.82	<0.001
Incidence of diabetes, *n* (%)	875 (6.1)	185 (6.5)	193 (6.8)	0.587	199 (7.0)	167 (5.8)	0.075	152 (5.3)	218 (7.6)	<0.001	162 (7.5)	166 (5.3)	<0.001
* Cohort time, years	4.64 ± 0.50	4.62 ± 0.53	4.63 ± 0.52	0.012	4.60 ± 0.58	4.66 ± 0.44	0.957	4.69 ± 0.39	4.55 ± 0.69	<0.001	4.53 ± 0.67	4.70 ± 0.34	<0.001
* Age, years	48.6 ± 11.0	51.3 ± 11.6	47.1 ± 10.3	<0.001	49.9 ± 11.0	48.5 ± 11.2	<0.001	47.2 ± 11.0	51.0 ± 11.0	<0.001	50.8 ± 10.9	47.5 ± 10.8	<0.001
^a^ Elders, *n* (%)	2572 (18.1)	738 (26.0)	384 (13.5)	<0.001	611 (21.6)	515 (18.2)	<0.001	450 (15.9)	682 (24.1)	<0.001	496 (23.2)	483 (15.5)	<0.001
Female, *n* (%)	7727 (54.5)	897 (31.6)	1842 (65.0)	<0.001	1025 (36.2)	1936 (68.3)	<0.001	1886 (66.5)	770 (27.2)	<0.001	831 (38.8)	2203 (70.6)	<0.001
Urban area, *n* (%)	4552 (32.1)	1069 (37.7)	632 (22.3)	<0.001	510 (18.0)	1296 (45.7)	<0.001	866 (30.5)	876 (30.9)	0.012	383 (17.9)	1363 (43.7)	<0.001
High school above, *n* (%)	2667 (18.8)	446 (15.7)	518 (18.3)	<0.001	257 (9.1)	742 (26.2)	<0.001	574 (20.2)	475 (16.8)	<0.001	176 (8.2)	871 (27.9)	<0.001
Household income ≥ 20,000 CNY/year, *n* (%)	3091 (21.8)	463 (16.3)	743 (26.2)	<0.001	401 (14.1)	770 (27.2)	<0.001	612 (21.6)	634 (22.4)	0.015	260 (12.1)	855 (27.4)	<0.001
^b^ Sufficient weekly physical activity, *n* (%)	12,623.3 (89.0)	2441.5 (86.1)	2579.4 (91.0)	<0.001	2516.3 (88.7)	2542.2 (89.7)	0.006	2580.7 (91.0)	2465.6 (86.9)	<0.001	1840.4 (85.9)	2818.1 (90.3)	<0.001
Current smoker, *n* (%)	3545 (25.0)	461 (16.3)	1102 (38.9)	<0.001	1081 (38.1)	435 (15.3)	<0.001	463 (16.3)	1426 (50.3)	<0.001	727 (33.9)	461 (14.8)	<0.001
Current alcohol drinker, *n* (%)	7817 (55.1)	1303 (46.0)	1901 (67.1)	<0.001	1636 (57.7)	1477 (52.1)	0.002	1342 (47.3)	2051 (72.3)	<0.001	1102 (51.4)	1722 (55.2)	0.008
Current tea drinker, *n* (%)	2684 (18.9)	426 (15.0)	703 (24.8)	<0.001	449 (15.8)	637 (22.5)	<0.001	202 (7.1)	1273 (44.9)	<0.001	376 (17.6)	641 (20.5)	0.112
^c^ Regular spicy food intake, *n* (%)	6465 (45.6)	1097 (38.7)	1570 (55.4)	<0.001	1361 (48.0)	6465 (45.6)	<0.001	1195 (42.2)	1628 (57.4)	<0.001	1002 (46.8)	1264 (40.5)	<0.001
* Total energy intake, kcal/day	1720.4 ± 660.4	1440.1 ± 609.2	2310.4 ± 706.1	<0.001	1820.2 ± 718.4	1910.3 ± 679.6	<0.001	1760.4 ± 688.0	2050.6 ± 726.8	<0.001	1740.1 ± 690.1	1720.5 ± 632.2	0.677
Protein, g/day	64.1 ± 32.1	41.5 ± 19.7	105.0 ± 36.6	<0.001	63.9 ± 35.2	77.0 ± 34.3	<0.001	65.7 ± 32.6	76.4 ± 36.1	<0.001	64.5 ± 34.0	65.5 ± 31.0	<0.001
Fat, g/day	65.3 ± 39.2	53.2 ± 35.7	92.0 ± 44.2	<0.001	63.3 ± 41.2	72.3 ± 38.9	<0.001	65.8 ± 40.7	73.0 ± 43.7	<0.001	73.8 ± 44.7	58.6 ± 33.3	<0.001
Carbohydrate, g/day	206 ± 91.4	194.0 ± 97.5	242.0 ± 102.0	<0.001	226.0 ± 102.0	232.0 ± 99.4	<0.001	223.0 ± 103.0	236.0 ± 99.7	<0.001	186.0 ± 89.8	225.0 ± 92.6	<0.001
Family history of diabetes, *n* (%)	978 (6.9)	181 (6.4)	203 (7.2)	0.792	164 (5.8)	247 (8.7)	<0.001	177 (6.2)	206 (7.3)	0.718	122 (5.7)	247 (7.9)	0.047
^d^ Overweight and above, *n* (%)	7338 (51.8)	1423 (50.2)	1563 (55.1)	0.001	1574 (55.5)	1342 (47.3)	<0.001	1289 (45.5)	1652 (58.3)	<0.001	1185 (55.3)	1471 (47.1)	<0.001
^e^ Abnormal serum creatinine levels, *n* (%)	677 (4.8)	150 (5.3)	132 (4.7)	0.352	150 (5.3)	139 (4.9)	0.502	145 (5.1)	127 (4.5)	0.183	94 (4.4)	147 (4.7%)	0.522
^e^ Abnormal systolic blood pressure, *n* (%)	2757 (19.4)	668 (23.6)	531 (18.7)	<0.001	684 (24.1)	453 (16.0)	<0.001	471 (16.6)	701 (24.7)	<0.001	576 (26.9)	465 (14.9%)	<0.001
^e^ Abnormal diastolic blood pressure, *n* (%)	1734 (12.2)	356 (12.6)	391 (13.8)	0.008	433 (15.3)	253 (8.9)	<0.001	259 (9.1)	486 (17.1)	<0.001	353 (16.5)	288 (9.2%)	<0.001
^e^ Abnormal triglyceride, *n* (%)	1254 (8.8)	253 (8.9)	284 (10.0)	0.172	271 (9.6)	274 (9.7)	0.062	237 (8.4)	303 (10.7)	0.012	235 (11.0)	244 (7.8)	<0.001
^e^ Abnormal total cholesterol, *n* (%)	1679 (11.8)	316 (11.1)	416 (14.7)	<0.001	421 (14.9)	261 (9.2)	<0.001	253 (8.9)	483 (17.0)	<0.001	257 (12.0)	299 (9.6)	<0.001
^e^ Abnormal low density lipoprotein cholesterol, *n* (%)	974 (6.9)	169 (6.0)	247 (8.7)	<0.001	236 (8.3)	147 (5.2)	<0.001	161 (5.7)	217 (7.7)	<0.001	162 (7.6)	173 (5.5)	0.006
^e^ Abnormal high density lipoprotein cholesterol, *n* (%)	616 (4.3)	113 (4.0)	150 (5.3)	0.157	133 (4.7)	135 (4.8)	0.512	126 (4.4)	139 (4.9)	0.600	103 (4.8)	119 (3.8)	0.340

The pattern score was divided into five points from low to high (Quintile1–Quintile5, Q1–Q5). ^†^ *p*-value is based on Student’s *t*-tests, Mann–Whitney U tests or Chi square tests as appropriate for two-sided testing, and there is *p* for trend based on a linear regression analysis for the variable of pattern score. * Pattern score, Cohort time, Age and Total energy intake are as numeric variables shown in Table 1, described by means (M) ± standard deviations (SDs). ^a^ Elders are defined as age at baseline ≥60 years old. ^b^ Sufficient weekly physical activity including work and leisure time is defined as ≥12.0 METs-h per week; METs, metabolic equivalents. ^c^ Regular spicy food intake is defined as eating spicy food ≥ 6 days per week for consideration of the prevalence and ritual preference of spicy food in Chongqing. ^d^ Overweight and above is defined as BMI ≥ 24 kg/m^2^ for there is few obese people (634, 4.5%). ^e^ Serum creatinine (SCr):male in normal: 20–59 years old is 57–97 μmol/L, 60–79 years old is 57–111 μmol/L; female in normal: 20–59 years old is 41–73 μmol/L, 60–79 years old is 41–81 μmol/L, beyond this range is abnormal; systolic blood pressure (SBP): 90–140 mmHg for normal, beyond this range is abnormal; diastolic blood pressure (DBP): 60–90 mmHg for normal, beyond this range is abnormal; triglyceride (TG): 0.56~1.70 mmol/L for normal, beyond this range is abnormal; total cholesterol (TC): 2.84~5.68 mmol/L for normal, beyond this range is abnormal; low density lipoprotein cholesterol (LDL-C): 2.10~3.10 mmol/L for normal, beyond this range is abnormal; high density lipoprotein cholesterol (HDL-C): 1.14~1.76 mmol/L for normal, beyond this range is abnormal.

**Table 2:** Hazard ratios(HR) between dietary patterns and diabetes using multi-level mixed effects cox models.

	Quintiles of Dietary Pattern Scores					*p* for Trend
	Q1 (Low)	Q2	Q3	Q4	Q5 (High)	
DASH pattern						
Cases	162	166	212	169	166	
Incidence rate (/1000 person/year)	2.46	2.52	3.22	2.57	2.52	
^a^ Model 1	1.00 (Reference)	0.86 (0.69, 1.06)	0.90 (0.74, 1, 11)	0.73 (0.59, 0.91) *	0.67 (0.54, 0.84) ***	<0.001
^b^ Model 2	1.00 (Reference)	0.88 (0.71, 1.09)	0.94 (0.77, 1.16)	0.77 (0.61, 0.96) *	0.71 (0.56, 0.90) **	0.002
^c^ Model 3	1.00 (Reference)	0.89 (0.72, 1.11)	0.96 (0.78, 1.19)	0.78 (0.62, 0.97) *	0.72 (0.57, 0.91) **	0.003
^d^ Model 4	1.00 (Reference)	0.92 (0.73, 1.14)	0.93 (0.75, 1.15)	0.80 (0.64, 1.00)	0.71 (0.40, 0.56) **	0.004
Alcohol-wheat products pattern						
Cases	152	135	163	207	218	
Incidence rate (/1000 person/year)	2.31	2.05	2.48	3.15	3.31	
^a^ Model 1	1.00 (Reference)	0.92 (0.73, 1.17)	1.10 (0.88, 1.38)	1.33 (1.08, 1.65) ***	1.41 (1.14, 1.75) ***	<0.001
^b^ Model 2	1.00 (Reference)	0.96 (0.76, 1.21)	1.16 (0.93, 1.46)	1.43 (1.16, 1.78) **	1.43 (1.15, 1.77) **	<0.001
^c^ Model 3	1.00 (Reference)	0.98 (0.78, 1.24)	1.20 (0.95, 1.50)	1.45 (1.17, 1.79) **	1.34 (1.06, 1.68) *	0.003
^d^ Model 4	1.00 (Reference)	0.98 (0.77, 1.25)	1.17 (0.93, 1.48)	1.50 (1.20, 1.86) ***	1.32 (1.04, 1.66) *	0.003
Meat pattern						
Cases	185	160	171	166	193	
Incidence rate (/1000 person/year)	2.81	2.43	2.60	2.52	2.93	
^a^ Model 1	1.00 (Reference)	0.89 (0.72, 1.11)	0.91 (0.74, 1.13)	0.87 (0.70, 1.08)	1.05 (0.85, 1.29)	0.792
^b^ Model 2	1.00 (Reference)	0.93 (0.75, 1.15)	0.95 (0.77, 1.17)	0.91 (0.73, 1.13)	1.09 (0.89, 1.35)	0.519
^c^ Model 3	1.00 (Reference)	0.92 (0.75, 1.14)	0.94 (0.76, 1.16)	0.90 (0.72, 1.11)	1.04 (0.82, 1.31)	0.983
^d^ Model 4	1.00 (Reference)	0.92 (0.74, 1.15)	0.93 (0.75, 1.15)	0.94 (0.75, 1.18)	1.02 (0.81, 1.30)	0.917
Dairy products-eggs pattern						
Cases	199	170	177	162	167	
Incidence rate (/1000 person/year)	3.03	2.58	2.69	2.46	2.54	
^a^ Model 1	1.00 (Reference)	0.89 (0.72, 1.09)	0.94 (0.77, 1.15)	0.86 (0.70, 1.07)	0.86 (0.70, 1.07)	0.179
^b^ Model 2	1.00 (Reference)	0.93 (0.75, 1.14)	0.98 (0.80, 1.21)	0.91 (0.73, 1.12)	0.94 (0.75, 1.17)	0.538
^c^ Model 3	1.00 (Reference)	0.92 (0.75, 1.14)	0.98 (0.80, 1.21)	0.89 (0.72, 1.11)	0.91 (0.72, 1.13)	0.355
^d^ Model 4	1.00 (Reference)	0.945 (0.77, 1.17)	0.943 (0.76, 1.17)	0.96 (0.77, 1.19)	0.91 (0.72, 1.14)	0.478

Quintile 1 is the reference category based on Cox proportional regression models. *: *p* < 0.05; **: *p* < 0.01; ***: *p* < 0.001. ^a^ Model 1 adjusted for sex and age (<60 or ≥60 years old). ^b^ Model 2 adjusted for region (urban or rural), educational level (primary school or below, Middle school or high school, high school above), household annual income (<20,000, 20,000–99,999, 10,000–19,999, ≥20,000 CNY/year), family history of diabetes, BMI (standard and below, overweight and above) + Model 1. ^c^ Model 3 adjusted for weekly physical activity (<12.0, ≥12.0 METs-h per week), smoking, drinking alcohol, drinking tea, eating spicy food (<1, 1–5, ≥6 days per week), daily total energy intake (kcal/day) + Model 2. ^d^ Model 4 adjusted for serum creatinine (Cr) (male in normal: 20–59 years old is 57–97 µmol/L, 60–79 years old is 57–111 µmol/L; female in normal: 20–59 years old is 41–73 µmol/L, 60-79 years old is 41–81 µmol/L, beyond this range is abnormal), systolic blood pressure (SBP) (90–140 mmHg for normal, beyond this range is abnormal), diastolic blood pressure (DBP) (60–90 mmHg for normal, beyond this range is abnormal), triglyceride (TG) (0.56~1.70 mmol/L for normal, beyond this range is abnormal), total cholesterol (TC) (2.84~5.68 mmol/L for normal, beyond this range is abnormal), low density lipoprotein cholesterol (LDL-C) (2.10~3.10 mmol/L for normal, beyond this range is abnormal), high density lipoprotein cholesterol (HDL-C) (1.14~1.76 mmol/L for normal, beyond this range is abnormal) + Model 3.

## Data Availability

Study data are available on request to the authors due to privacy and legal reason.

## Supplementary Materials

The following supporting information can be downloaded at: https://www.mdpi.com/article/10.3390/nu16111636/s1, Figure S1: The distribution of food intake in quintiles in each patterns; Table S1: Diet-related questionnaire (excerpt from the CMEC questionnaire); Table S2: Sociodemographic characteristics and lifestyle-related questionnaires (excerpt from the CMEC questionnaire); Table S3: Total energy intake calculation method in CMEC; Table S4: The reproducibility and validity assessment of food frequency questionnaire (FFQ); Table S5: The scoring criterion for DASH pattern; Table S6: Adjusted hazard ratios (HR) in each dietary pattern in the subgroup analysis. References [19,41,42,43,44,45,46,47,48,49] are cited in the Supplementary Materials.
