# Supplementary material for: Dietary Patterns and New-Onset Diabetes Mellitus in Southwest China: A Prospective Cohort Study in the China Multi-Ethnic Cohort (CMEC)
Source: Nutrients. 2024 May 27;16(11):1636. doi: 10.3390/nu16111636 (PMC11174084; doi:10.3390/nu16111636)
Supplement: Supplementary file 1 [file nutrients-16-01636-s001.zip › nutrients-3004130-supplementary.pdf]

Supplementary

Table S1. Diet-related questionnaire (excerpt from the CMEC questionnaire)

**Alcohol consumption**			
A1	**Have you drunk any alcohol during past 24 hours?**		
□ Yes	□ No		
A2	**During the past 12 months, how often did you drink any alcohol?**		
□ Never			→ jump to next section
□ Only occasionally			
□ Only at certain seasons			
□ Every week but less than weekly			
□ At least once a week			→ jump to A4
A3a	**In the past, have you ever drunk every week for at least one year?**		
□ Yes			→ jump to A3b
□ No			→ jump to next section
A3b	**How long have you stopped this behavior (drunk every week for at least one year) ?**		
□ □ years			→ jump to next section
A4	**During the past 12 months, on how many days did you drink alcohol in a typical week?**		
□ 1-2 days/week			
□ 3-5 days/week			
□ Daily/almost everyday			
A5	**When did you start drinking some alcohol in most weeks?**		
□ years old			
A6	**On three different situations, what kind(s) of alcoholic drinks you choose and how much you usually drink in a day?** （Can choose up to 3 types of alcohol for special occasions）		
	Typical occasion (choose one)	On a special day when you drink a lot	Last time when you drink
Beer	□ □ Bottle/week	□ □ Bottle/day	□ □ Bottle/day
rice wine /fruit wine （<10°）	□ □ *liang/week	□ □ *liang /day	□ □ *liang /day
Highland barley wine	□ □ *liang /week	□ □ *liang /day	□ □ *liang /day
Wine	□ □ *liang /week	□ □ *liang /day	□ □ *liang /day
Spirit or Chinese baijiu （≥40°）	□ □ *liang /week	□ □ *liang /day	□ □ *liang /day
Spirits or Chinese baijiu (<40°)	□ □ *liang /week	□ □ *liang /day	□ □ *liang /day
Rice wine (30°-40°）	□ □ *liang /week	□ □ *liang /day	□ □ *liang /day
* liang ： This is one of the mass units being used in modern China. 1 liang = 50 g			
A7	**On a typical day when you drink alcohol, when do you usually take the drink?**		
□ Usually before meals			
□ Drink with meals			
□ Usually drink between or after meals			
□ Usually before sleep			

□ No regular pattern

A8	**After drinking alcohol, do you usually experience hot flushes or dizziness?**

□ Yes, soon after first mouthful

□ Yes, after drinking small amount of alcohol

□ Yes, but only after drinking large amount of alcohol

□ No

A9	**During the past month, have you ever had the following experience?**

	Yes	No
Unable to work or to do anything because of drinking	□	□
Felt depressed, angry or couldn't control yourself after drinking	□	□
Could not keep away from drinking	□	□
Had shaken when you stopped drinking	□	□

A10	**Has your alcohol consumption changed significantly compared with that some years ago?**

□ About same as before

□ Increased significantly

□ Decreased significantly

Tea and Beverages

1 Tea

B1	**Have you ever drunk tea weekly and last over half year?**

□ Yes

□ No → jump to B9

B2	**When did you start drinking tea in most weeks?**

□ years old

B3	**During the past 12 months, how many days did you drink tea in a typical week?**

□ Does not drink tea currently → jump to B3a since □ □ year old stop drinking

□ 1-2 day(s)/week

□ 3-5 days/week

□ Daily/almost everyday

B4	**On days when you drink, which type of tea do you prefer, how many cups do you usually drink?** （choose one only）

Green tea	□ □ cups/day
Jasmine	□ □ cups/day
Dark tea (brick tea, Pu-erh tea, etc.)	□ □ cups/day
Sweety tea (milk+ black tea)	□ □ cups/day
Black tea	□ □ cups/day
Oolong tea (Tie Guanyin tea)	□ □ cups/day
Yellow tea (Mengding huaya tea)	□ □ cups/day
White tea (Yinzhen tea)	□ □ cups/day

B5	**How often do you change tea leaves during a day?**

□ □ times

B6	**How much tea leaves do you usually add each time?**

□ □ grams

B7	**What strength of tea do you usually prefer to drink?**		
□ Weak	□ Moderate	□ Strong	
B8	**What temperature do you usually drink your tea?**		
□ Bunning hot		□ Hot	□ Room temperature / warm
2	Beverages		
B9	**Have you ever drunk every week and last over half year?**		
□ Yes			
□ No			→ jump to next section
B10	**How old did you start to develop the habit of drinking beverages?**		
□ □ years old			
B11	**How many days did you drinking beverages in a typical week during the past 12 months?**		
□ Don't drink currently		→ jump to B11a	since □ □ year old stop drinking
□ 1-2 day(s)/week			
□ 3-5 days/week			
□ Daily/almost everyday			
B12	**What kind of drink do you drink most often and what is the frequency?** (choose one)		
Sweeten beverage		□ □ cups/week	
Coffee and caffeine beverage		□ □ cups/week	
Others		□ □ cups/week	

Diets

C1		
C1a	How many people usually eat breakfast together in your family during the past month?	□ □
C1b	How many people usually eat lunch together in your family during the past month?	□ □
C1c	How many people usually eat dinner together in your family during the past month?	□ □
C2	**What kind of oil be used mostly in your family?** （choose at most two）	
□ Rapeseed oil/Sesame oil	kg/month	
□ Peanut oil	kg/month	
□ Soybean oil	kg/month	
□ Lard oil	kg/month	
□ Blended oil	kg/month	
□ Others	kg/month	
C3	**How many grams salt does your family usually consume per month?**	
□ grams/month		

During the past 12 months, how often did you consume the following foods and how much the weight per weight (For individual)!

		a Eat or not		b Frequency				c Weight per time	
		Yes	No	b1 times/d	b2 times/w	b3 times/m	b4 times/y	Uncooked	Cooked
C4	Rice	□	□	□	□	□	□	—	□ g
C5	Wheat products	□	□	□	□	□	□	—	□ g
C6	Coarse grain	□	□	□	□	□	□	□ g	—
C7	Tubers	□	□	□	□	□	□	□ g	—

C8	Meat	□	□	□	□	□	□	□ g	—
C9	Poultry	□	□	□	□	□	□	□ g	—
C10	Fish/sea food	□	□	□	□	□	□	□ g	—
C11	Eggs	□	□	□	□	□	□	□ g	—
C12	Fresh vegetables	□	□	□	□	□	□	□ g	—
C13	Soybean products	□	□	□	□	□	□	□ g	—
C14	Preserved vegetables	□	□	□	□	□	□	□ g	—
C15	Fresh fruit	□	□	□	□	□	□	□ g	—
C16	Dairy products	□	□	□	□	□	□	□ g	—

C17	**During the past 12 months, have you taken dietary supplements regularly last over a month?**
□ Yes	
□ No	

C18	**What kind of dietary supplements have you ever taken?**	
C18a Fish oil/cod liver oil	□ Yes	□ No
C18b Vitamin D	□ Yes	□ No
C18c Other vitamins	□ Yes	□ No
C18d Calcium pills	□ Yes	□ No
C18e Others	□ Yes	□ No

Spicy food

H23	**How often did you eat spicy food in the past month?**	
□	Never/hardly ever eat	→ jump to H27
□	A few times, but less than once a week	→ jump to H27
□	Eat spicy food 1-2 days a week	
□	Eat spicy food 3-5 days a week	
□	Eat every day or almost every day	

H24	**How old were you when you started eating spicy food on a weekly basis?**
□ □ years old	

H25	**Do you usually eat mild, spicy or very spicy food?**
□	Mild spicy
□	Spicy
□	Very spicy

H26	**What spicy ingredients do you usually use when you eat spicy food?** (Multiple choices possible)
□	Chili sauce
□	Chili oil
□	Dried pointy red pepper
□	Fresh sharp chili
□	Others (e.g. curries or spicy spices)

Table S2. Sociodemographic characteristics and lifestyle-related questionnaires (excerpt from the CMEC questionnaire)

Respondent name:
Home Address: __County (city/district) __Township (street) __Village (Neighborhood Committee) ________________ (Detailed address)
Individual code:□□□□
Investigator Signature: ____ Date: ____year _____month____ day

Sociodemographic characteristics

A1 Sex	□ Male	□ Female
A3 Id number □□□□□□□□□□□□□□□□□□		
A4 **Your current registered permanent residence status is**		
□ Agricultural household registration	□ Non-agricultural household	
□ Unified household registration	□ Have no household registration	
A6 **The highest level of education you have received is**		
□ Never went to school	□ Primary school	
□ Junior high school	□ Senior secondary (including technical/technical schools)	
□ Junior college	□ University (including postgraduate and above)	
A8		
A8a **Whether you are enrolled in the following medical insurance**		
□ Basic medical insurance for urban workers (including full public medical insurance)		
□ Basic medical insurance for urban residents		
□ New rural cooperative medical care		
□ Basic medical insurance for urban and rural residents		
□ No above medical insurance		

A9 **How many members of your family live together, including yourself?** N=______________

A13 **How much was your family's total income in the past year (before tax)?**

□ <12000 CNY

□ 12000-19999 CNY

□ 20000-59999 CNY

□ 60000-99999 CNY

□ 100000-199999 CNY

□ ≥200000 CNY

Lifestyle-related

B1 **Do you smoke?**

□ Never

□ Current smoker

□ Have quit smoking (quit smoking for more than half a year)

1. Physical Activity - non-agricultural workers

F1 **In the past year, did you sit or stand at work or did you do most of your physical work?**

□ Sit-in (e.g., executive, secretary, etc.) → jump to F1a: Average weekly sitting □□□ hours

□ Mainly standing (e.g. salespeople, doormen, etc.)

□ General physical work (do not sweat too much at room temperature, such as plumber, electrician, woodworker, mason, etc.)

□ Heavy manual labor (easy to sweat at room temperature, such as loading and unloading, mining, steelmaking, etc.)

□ Retirement or housework or unemployed for more than one year or physical disability cannot work normally → jump to F13

F2 **How many hours do you work per week on average?** □□□hours

F3 **In the past year, how did you usually get to and from work?** (single choice)

□ On foot

□ Ride a motorcycle/scooter

□ Ride a bike

□ Private or public transport (car, subway, ferry)

□ Usually work from home or near home → jump to F13

F4 **How long do you usually spend commuting to and from work?**
□□□minutes

2. **Physical activity - Agricultural and pastoral workers**

F5 **Are there distinct gaps in your production activities?**

□ Yes

□ No → jump to F7

F6

F6a **How long was the cumulative duration of the busy season in the past year?**
□□months

F6b **During the busy farming season in the past year, how did you usually do your work?**

□ Man-oriented

□ Semi-mechanized

□ Mainly mechanized

F6c **During the busy season of the past year, how many hours of farm work did you do in an average day?**
□□.□hours

F6d **During the busy farming season in the past year, how many hours per day did you sweat or feel your heart rate increase due to heavy work?**
□□.□hours

F7 **How many hours do you do farm work per week, usually or not?** □□□hours/a week

F8 **Do you do any other work besides farm work?**

□ Yes

□ No → jump to F11

F9 **What other jobs do you have in which you do most of your work sitting or standing or doing heavy physical work?**

□ Mainly meditate (knitting, sewing, etc.) → jump to: F9a average weekly sitting □□□hours

□ Mainly standing (e.g. salespeople, doormen, etc.)

□ General manual work (carpentry, electrician, construction, etc.)

□ Heavy manual labor (porters, miners, stevedores, etc.)

F10 **In addition to farm work, how many cumulative hours do you usually do other work per week?** □□□hours

F11 **In the past year, how did you usually work outside the home? (single choice)**

□ On foot

□ Ride a motorcycle/scooter

□ Ride a bike

□ Private or public transport (car, subway, ferry)

□ Usually at home → jump to F13

F12 **How long do you usually spend commuting to and from work?** □□□minutes

3. Physical activity - Shared section

F13 **In the past year, how often did you take part in physical exercise in your spare time**？

□ Never or hardly ever → jump to F16

□ 1-3 times a month → jump to F16

□ 1-2 times a week

□ 3-5 times a week

□ Exercise every day or almost every day

F14 **If you exercise every week, what is the most common type of exercise?**

□ Tai Chi/Qigong/Walking

□ Fast walking/health exercises/Yangko/square dance

□ Running/aerobics

□ Swimming

□ Ball games (basketball, table tennis, badminton, etc.)

□ Exercise with equipment

□ Others (e.g. mountain climbing)

F15 **In the past year, how many hours per week have you participated in leisure sports?** □□□ hours/a week

F16 **In the past year, on average, how many days per week did you sweat or have a significantly increased heart rate as a result of intense physical activity such as exercise/work?**

□ Never or almost never → jump to F18

□ Sometimes, but not every week → jump to F18

□ One or two days a week

□ Three to five days a week

□ Three to five days a week

F17 **On average, how many cumulative hours per week do you engage in similar strenuous physical activities as described**

above? □□hours/a week

F18 **On average, how many hours per week do you spend doing various types of housework (including childcare)?**

□□□hours/a week

F19 **How much time do you spend sitting in your spare time each week on average (such as playing chess/mobile phone/tablet/ watching TV/reading/knitting, etc.)?**

□□□hours/a week

G5 **How old were you at the time of each birth and how was your child breastfed after birth?** (Birth of twins counts once)

Number of live births	G5a Age at childbearing	G5b Breastfeeding cycle
1	□□years old	Breastfeed for□□months
2	□□years old	Breastfeed for□□months
3	□□years old	Breastfeed for□□months
4	□□years old	Breastfeed for□□months
5	□□years old	Breastfeed for□□months
------------	------------	------------
15	□□years old	Breastfeed for□□months

Table S3. Total energy intake calculation method in CMEC

Since the FFQ used in the CMEC study only includes 13 crude food groups rather than specific food items, we were not able to calculate the total energy intake based on the exact energy of each food item. Instead, we used the energy intake standard which had published [19,41–43] to conduct our study. On account of the 3.1% response rate in beverages (such as coffee, sugar-sweetened beverages), 18 kinds of food (Rice, wheat products, coarse grain, tubers, red and processed meats, poultry, fish/sea food, eggs, fresh vegetables, soybean products, preserved vegetables, fresh fruits, dairy products, alcohol, vegetable oil, animal oil, salt and tea) were included for establishing the posterior dietary pattern. In addition to individual inquiries about food, the amounts of oil and salt reported for the entire family were divided by the number of family members and then divided by the proportion of meals consumed at home and converted to daily intake.

Food Category	Weight (g)	Energy (kcal)	Protein (g)	Fat (g)	Carbohydrate (g)
Rice	77	90	2.0	0.2	19.4
Wheat products	35	90	2.5	0.4	18.0
Coarse grain	25	90	2.5	0.7	18.0
Tubers	100	90	1.9	0.2	20.0
Red and processed meats	100	323	27.5	23.2	1.1
Poultry	50	90	8.8	6.0	0.7
Fish/sea food	80	90	14.8	2.9	1.7
Eggs	60	90	7.6	6.6	1.6
Fresh vegetables	450	90	4.5	0.7	16.0
Soybean products	25	90	5.5	0.5	15.0
Preserved vegetable	300	90	6.6	0.8	17.0
Fresh fruits	200	90	1.0	0.6	20.0
Dairy products	160	90	5.0	5.4	7.4
Alcohol	1	7	0	0	0
Vegetable oil	100	899	0	99.9	0
Animal oil	100	897	0	99.7	0

References

19. Xiao, X.; Qin, Z.; Lv, X.; Dai, Y.; Ciren, Z.; Yangla, Y.; Zeng, P.; Ma, Y.; Li, X.; Wang, L.; et al. Dietary patterns and cardiometabolic risks in diverse less-developed ethnic minority regions: Results from the China Multi-Ethnic Cohort (CMEC) Study. Lancet Reg. Health West. Pac. 2021, 15, 100252. https://doi.org/10.1016/j.lanwpc.2021.100252.

41. WS/T 429-2013; Dietary Guide for Adult Diabetes Patients. National Health Commission of the People's Republic of China: Beijing, China, 2013.

42. National Institute for Nutrition and Health. China Food Composition Tables, 6 ed.; Peking University Medical Press: Beijing, China, 2018.

43. National Bureau of Statistics. China Statistical Yearbook—2018; China Statistics Press: Beijing, China, 2019.

Table S4. The reproducibility and validity assessment of food frequency questionnaire (FFQ).

In a subsample of 3,742 participants in Chongqing, the FFQ was repeated one year after the baseline survey from August 2020 to November 2020 to assess its reproducibility and validity. We matched the participants with a unique code, finally included 2344 participant for assessment. Then we used statistical methods of Interclass Correlation Coefficient (ICC) and Spearman correlation to check the reliability of dietary information. Food group intakes were log-transformed before calculating the ICC and Spearman coefficients. On the whole, the factors' validation was general in reliability and moderate in validity.

Food Category	ICC	Spearman coefficients
Rice	0.25	0.60
Wheat products	0.53	0.62
Coarse grain	0.52	0.56
Tubers	0.52	0.56
Red meat	0.55	0.57
Poultry	0.58	0.65
Fish/Sea food	0.60	0.60
Eggs	0.43	0.48
Fresh vegetables	0.23	0.41
Soybean products	0.45	0.44
Preserved vegetables	0.62	0.62
Fresh fruits	0.50	0.56
Dairy products	0.52	0.63
Alcohol	0.48	0.52
Tea	0.68	0.65
Vegetable oil	0.55	0.56
Animal oil	0.45	0.54
Salt	0.50	0.56

Table S5. The scoring criterion for DASH pattern.

To assess the adherence to the one of most well-studied priori dietary pattern, we used the modified DASH score1,2 with slight adaption according to the CEMC data. We didn't included other priori dietary patterns like Mediterranean diet pattern and Plant-based dietary pattern, and the reasons are that, firstly, the published relevant studies [19,45] in CMEC emphasized the significance of DASH pattern; secondly, we have tried to form the two patterns by the international standards [44,46–49], however, the results were not statistically significant; lastly, the main purpose of our research is to identify the regional dietary patterns, and used the DASH pattern to instruct their dietary patterns.

Component	FFQ item	Foods in details	Scoring criteria	Q1, g/day	Q5, g/day
Fruit	C15	All fresh fruit	Q1=1 point Q2=2 points Q3=3 points Q4=4 points Q5=5 points	28.6	200.0
Vegetable	C12	All fresh vegetables except tubers and legumes		200.0	500.0
Legumes	C13	Soybeans, black beans, tofu, soybean milk, dried beans, dried bean curd, etc.		0.0*	14.3
Dairy Product	C16	Fresh milk, yogurt, cheese, milk tea, low-fat dairy, etc.		0.0*	142.9
Whole Grains	C6	Oats, sorghum, dried corn, highland barely, etc.		0.0*	26.2
Red & processed meat	C8	Beef, mutton, pork and their products	Reverse score: Q1=5 points Q2=4 points Q3=3 points Q4=2 points Q5=1 point	28.6	100.0
Sodium	C1, C3, C14	Sodium in salt and preserved vegetables		1.1	6.4

* '0' represented for the people who have not eaten this type of food in the past year.

References

19. Xiao, X.; Qin, Z.; Lv, X.; Dai, Y.; Ciren, Z.; Yangla, Y.; Zeng, P.; Ma, Y.; Li, X.; Wang, L.; et al. Dietary patterns and cardiometabolic risks in diverse less-developed ethnic minority regions: Results from the China Multi-Ethnic Cohort (CMEC) Study. Lancet Reg. Health

West. Pac. 2021, 15, 100252. https://doi.org/10.1016/j.lanwpc.2021.100252.
44. Fung, T.T., Chiuve, S.E., McCullough, M.L., Rexrode, K.M., Logroscino, G., Hu, F.B. Adherence to a DASH-Style Diet and Risk of Coronary Heart Disease and Stroke in Women. Arch. Intern. Med. 2008, 168, 713–720. https://doi.org/10.1001/archinte.168.7.713.
45. Chen, L.; Tang, W.; Wu, X.; Zhang, R.; Ding, R.; Liu, X.; Tang, X.; Wu, J.; Ding, X. Eating Spicy Food, Dietary Approaches to Stop Hypertension (DASH) Score, and Their Interaction on Incident Stroke in Southwestern Chinese Aged 30–79: A Prospective Cohort Study. Nutrients 2023, 15, 1222. https://doi.org/10.3390/nu15051222.
46. Xu, H., Guo, B., Qian, W., Ciren, Z., Guo, W., Zeng, Q.; Mao, D.; Xiao, X.; Wu, J.; Wang, X.; et al. Dietary Pattern and Long-Term Effects of Particulate Matter on Blood Pressure: A Large Cross-Sectional Study in Chinese Adults. Hypertension 2021, 78, 184–194. https://doi.org/10.1161/HYPERTENSIONAHA.121.17205.
47. Bonaccio, M., Di Castelnuovo, A., Costanzo, S., Gialluisi, A., Persichillo, M., Cerletti, C.; Donati, M.B.; de Gaetano, G.; Iacoviello, L.. Mediterranean diet and mortality in the elderly: A prospective cohort study and a meta-analysis. Br. J. Nutr. 2018, 120, 841–854. https://doi.org/10.1017/S0007114518002179.
48. Baden, M.Y., Liu, G., Satija, A., Li, Y., Sun, Q., Fung, T.T.; Rimm, E.B.; Willett, W.C.; Hu, F.B.; Bhupathiraju, S.N. Changes in Plant-Based Diet Quality and Total and Cause-Specific Mortality. Circulation 2019, 140, 979–991. https://doi.org/10.1161/CIRCULATIONAHA.119.041014.
49. Chiu, S., Bergeron, N., Williams, P.T., Bray, G.A., Sutherland, B., Krauss, R.M. Comparison of the DASH (Dietary Approaches to Stop Hypertension) diet and a higher-fat DASH diet on blood pressure and lipids and lipoproteins: A randomized controlled trial. Am. J. Clin. Nutr. 2016, 103, 341–347.

Figure S1. The distribution of food intake in quintiles in each pattern.

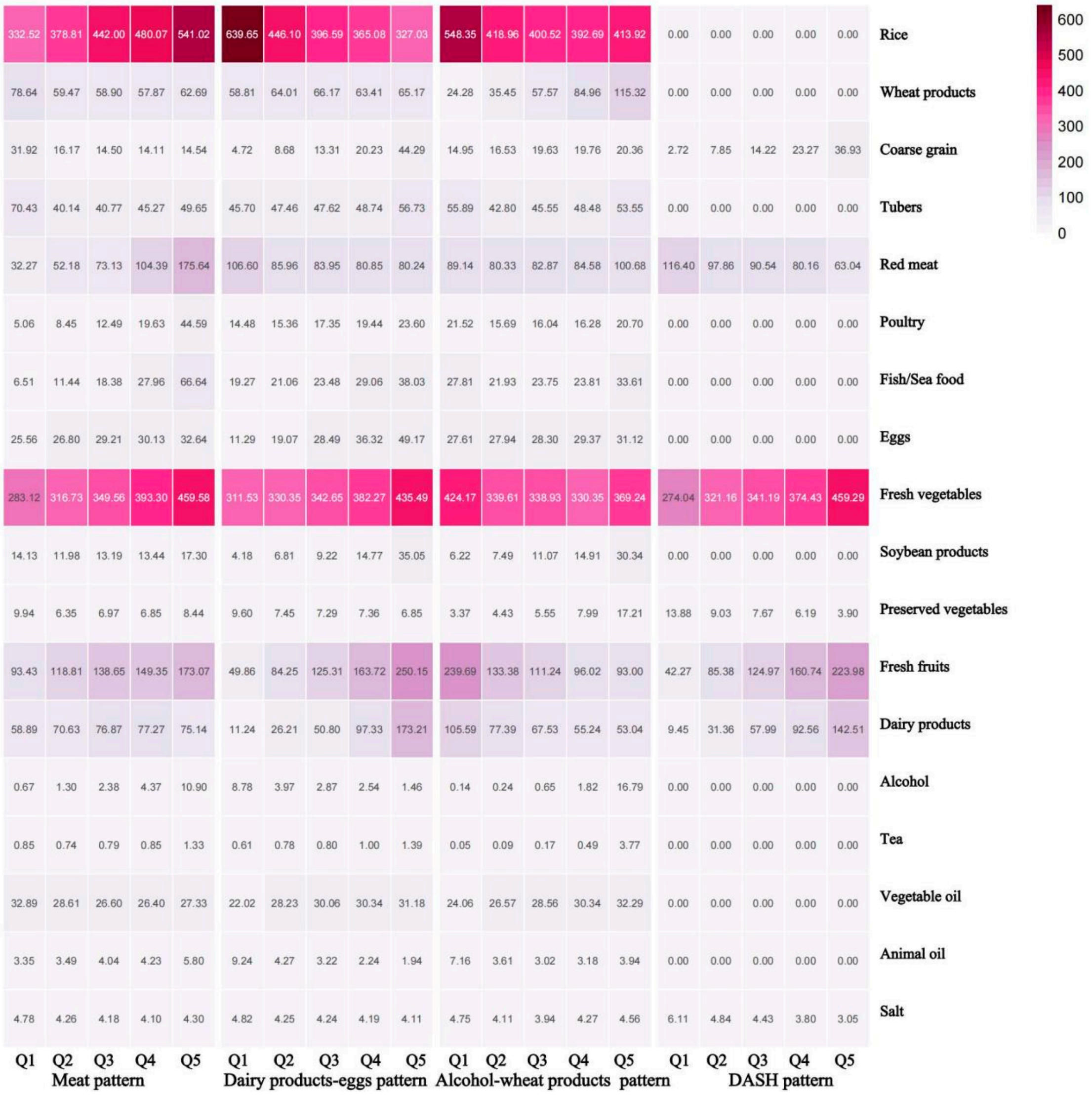

showed the distribution of 18 food categories' daily intake (g/day) in quintiles in each pattern. Red indicates the daily intake increased, white indicates the daily intake decreased. Due to unadjusted food intake, the daily intake ranges from 0 to 640.0 g/day. Q1, Q2, Q3, Q4 and Q5 was defined based on quintiles.

Table S6. Adjusted hazard ratios (HR) in each dietary pattern in the subgroup analysis.

Table S6A. Adjusted hazard ratios (HR) in DASH pattern and Alcohol-wheat products pattern in the subgroup analysis[1].

**Subgroup**		**DASH pattern**					***P* for interaction**	**Alcohol-wheat products pattern**					***P* for interaction**
		**Q1**	**Q2**	**Q3**	**Q4**	**Q5**		**Q1**	**Q2**	**Q3**	**Q4**	**Q5**	
Sex	Female	1.00	**0.50(0.35-0.72)**	**0.64(0.46-0.89)**	**0.52(0.37-0.73)**	**0.40(0.28-0.57)**	**0.002**	1.00	1.02(0.72-1.46)	1.15(0.82-1.61)	1.37(1.00-1.86)	1.21(0.89-1.65)	0.958
	Male	1.00	1.16(0.88-1.53)	1.14(0.87-1.50)	0.92(0.68-1.24)	0.99(0.72-1.35)		1.00	0.95(0.69-1.30)	1.19(0.87-1.61)	**1.5(1.11-2.04)**	1.42(0.97-2.06)	
Age	younger	1.00	0.89(0.68-1.17)	0.99(0.77-1.28)	**0.71(0.53-0.93)**	**0.69(0.52-0.92)**	0.520	1.00	1.02(0.76-1.36)	**1.35(1.03-1.78)**	**1.57(1.20-2.04)**	**1.49(1.12-1.98)**	0.456
	elder	1.00	0.82(0.56-1.2)	0.86(0.59-1.25)	0.93(0.63-1.36)	0.76(0.51-1.14)		1.00	0.84(0.55-1.27)	0.89(0.60-1.34)	1.12(0.77-1.63)	1.04(0.70-1.53)	
BMI (kg/m²)	<24	1.00	0.73(0.47-1.12)	0.95(0.64-1.42)	0.65(0.42-1.01)	**0.60(0.38-0.94)**	0.769	1.00	1.05(0.75-1.48)	1.08(0.76-1.51)	1.33(0.95-1.84)	1.37(0.96-1.95)	0.364
	≥ 24	1.00	0.94(0.73-1.21)	0.94(0.74-1.21)	0.81(0.62-1.06)	**0.73(0.55-0.97)**		1.00	0.89(0.64-1.23)	1.24(0.92-1.67)	**1.49(1.12-1.99)**	1.28(0.94-1.73)	
Region	Urban	1.00	1.03(0.64-1.67)	1.27(0.81-1.98)	1.06(0.68-1.66)	0.89(0.56-1.41)	0.234	1.00	1.03(0.69-1.53)	1.54(1.06-2.25)	1.32(0.89-1.96)	1.49(0.99-2.26)	0.160
	Rural	1.00	0.84(0.65-1.08)	0.85(0.67-1.09)	**0.64(0.49-0.85)**	**0.64(0.47-0.85)**		1.00	0.97(0.72-1.30)	1.03(0.78-1.37)	**1.51(1.17-1.96)**	1.26(0.95-1.66)	
Family history of diabetes	No	1.00	0.88(0.69-1.11)	0.94(0.75-1.17)	0.84(0.66-1.06)	**0.72(0.56-0.93)**	0.054	1.00	1.03(0.8-1.31)	1.17(0.92-1.49)	**1.44(1.14-1.81)**	**1.36(1.07-1.74)**	0.476
	Yes	1.00	0.97(0.51-1.84)	1.09(0.58-2.04)	0.34(0.15-0.77)	0.63(0.32-1.27)		1.00	0.68(0.31-1.53)	1.42(0.72-2.78)	1.68(0.85-3.29)	1.40(0.69-2.83)	
Current smokers	No	1.00	0.82(0.63-1.08)	0.85(0.65-1.11)	**0.74(0.56-0.98)**	**0.61(0.46-0.81)**	0.127	1.00	1.00(0.76-1.30)	1.25(0.97-1.62)	**1.47(1.14-1.90)**	**1.34(1.00-1.80)**	0.795
	Yes	1.00	0.98(0.68-1.41)	1.16(0.82-1.65)	0.84(0.57-1.25)	1.01(0.67-1.52)		1.00	0.83(0.49-1.41)	0.89(0.55-1.44)	1.28(0.84-1.94)	1.06(0.71-1.59)	
Current alcohol drinkers	No	1.00	0.63(0.46-0.87)	0.76(0.56-1.02)	**0.66(0.48-0.92)**	**0.46(0.32-0.66)**	**0.036**	1.00	0.97(0.70-1.35)	**1.36(1.00-1.86)**	**1.61(1.19-2.19)**	**1.51(1.05-2.17)**	0.567
	Yes	1.00	1.19(0.87-1.62)	1.20(0.89-1.62)	0.90(0.66-1.25)	0.99(0.72-1.38)		1.00	0.95(0.67-1.34)	0.98(0.71-1.37)	1.26(0.93-1.72)	1.11(0.82-1.51)	

Spicy food(days/week)	<1	1.00	0.7(0.45-1.08)	1.04(0.68-1.58)	0.93(0.59-1.46)	0.78(0.49-1.24)	0.592	1.00	0.92(0.60-1.40)	1.03(0.68-1.56)	1.08(0.71-1.64)	0.82(0.50-1.33)	0.129
	1-5y	1.00	0.88(0.55-1.4)	0.80(0.52-1.23)	**0.57(0.36-0.91)**	**0.57(0.36-0.90)**		1.00	1.12(0.73-1.73)	1.29(0.85-1.96)	1.48(0.98-2.23)	**1.91(1.24-2.97)**	
	≥6	1.00	0.96(0.7-1.30)	0.98(0.72-1.32)	0.78(0.56-1.08)	**0.70(0.49-0.99)**		1.00	0.99(0.67-1.45)	1.34(0.93-1.91)	**1.72(1.23-2.39)**	**1.44(1.02-2.03)**	

Table S6B. Adjusted hazard ratios (HR) in Meat pattern and Dairy products-eggs pattern in the subgroup analysis[1].

Subgroup		Meat pattern					*P* for interaction	Dairy products-eggs pattern					*P* for interaction
		**Q1**	**Q2**	**Q3**	**Q4**	**Q5**		**Q1**	**Q2**	**Q3**	**Q4**	**Q5**	
Sex	Female	1.00	0.84(0.63-1.12)	0.86(0.64-1.16)	0.79(0.57-1.1)	0.80(0.54-1.17)	0.440	1.00	0.74(0.53-1.04)	0.75(0.53-1.04)	**0.70(0.50-0.98)**	**0.60(0.42-0.85)**	0.063
	Male	1.00	1.07(0.77-1.49)	1.03(0.75-1.42)	1.04(0.77-1.42)	1.23(0.89-1.68)		1.00	0.99(0.76-1.30)	1.13(0.87-1.48)	0.99(0.75-1.32)	1.17(0.87-1.57)	
Age	younger	1.00	0.85(0.65-1.11)	0.89(0.68-1.16)	0.9(0.69-1.18)	0.99(0.74-1.32)	0.656	1.00	0.86(0.67-1.10)	0.90(0.7-1.16)	**0.75(0.57-0.98)**	0.84(0.64-1.11)	0.160
	elder	1.00	1.05(0.74-1.48)	1.04(0.73-1.48)	0.86(0.59-1.26)	1.10(0.74-1.64)		1.00	1.01(0.69-1.49)	1.16(0.8-1.69)	1.26(0.87-1.83)	1.02(0.68-1.51)	
BMI (kg/m²)	<24	1.00	0.87(0.59-1.26)	1.10(0.76-1.6)	0.91(0.61-1.37)	1.23(0.80-1.90)	0.725	1.00	0.88(0.59-1.31)	0.82(0.54-1.25)	0.85(0.56-1.3)	0.84(0.55-1.29)	0.827
	≥ 24	1.00	0.95(0.73-1.23)	0.88(0.68-1.14)	0.90(0.69-1.16)	0.95(0.72-1.26)		1.00	0.96(0.75-1.22)	1.05(0.83-1.34)	0.90(0.70-1.17)	0.92(0.70-1.20)	
Region	Urban	1.00	1.06(0.76-1.5)	1.05(0.75-1.48)	0.77(0.52-1.13)	1.06(0.69-1.61)	0.292	1.00	1.19(0.75-1.90)	1.37(0.88-2.13)	1.09(0.69-1.71)	1.35(0.87-2.11)	0.129
	Rural	1.00	0.84(0.64-1.11)	0.87(0.66-1.14)	0.95(0.73-1.24)	1.02(0.77-1.35)		1.00	0.87(0.69-1.10)	0.88(0.68-1.12)	0.85(0.66-1.10)	**0.73(0.55-0.96)**	
Family history of diabetes	No	1.00	0.9(0.72-1.13)	0.91(0.73-1.14)	0.89(0.71-1.13)	1.04(0.81-1.33)	0.925	1.00	0.89(0.71-1.11)	1.00(0.80-1.24)	0.89(0.71-1.12)	0.95(0.75-1.21)	0.321
	Yes	1.00	1.05(0.55-2.02)	1.17(0.63-2.17)	0.91(0.48-1.76)	0.99(0.49-2.01)		1.00	1.17(0.64-2.17)	0.87(0.45-1.69)	0.84(0.44-1.59)	0.61(0.30-1.21)	
Current smokers	No	1.00	0.86(0.67-1.09)	0.94(0.74-1.19)	0.71(0.55-0.94)	0.91(0.67-1.22)	0.002	1.00	0.92(0.71-1.20)	0.89(0.68-1.16)	0.89(0.68-1.17)	0.82(0.62-1.09)	0.181
	Yes	1.00	1.40(0.86-2.28)	1.20(0.75-1.92)	1.53(0.99-2.38)	1.45(0.92-2.27)		1.00	0.89(0.63-1.26)	1.19(0.85-1.67)	0.85(0.59-1.24)	1.18(0.81-1.72)	

Current alcohol drinkers	No	1.00	0.85(0.64-1.13)	0.84(0.62-1.12)	0.82(0.6-1.12)	0.88(0.61-1.27)	0.219	1.00	0.91(0.67-1.23)	0.95(0.70-1.3)	0.76(0.54-1.06)	0.81(0.58-1.13)	0.731
	Yes	1.00	1.05(0.76-1.46)	1.09(0.80-1.50)	1.02(0.74-1.39)	1.17(0.84-1.61)		1.00	0.92(0.69-1.22)	1.01(0.77-1.34)	1.02(0.76-1.35)	0.97(0.72-1.31)	
Spicy food(days/week)	<1	1.00	1.04(0.7-1.54)	0.91(0.59-1.42)	0.85(0.54-1.35)	1.55(0.96-2.51)	0.552	1.00	0.88(0.58-1.35)	0.99(0.64-1.54)	1.04(0.68-1.60)	0.87(0.55-1.38)	0.866
	1-5y	1.00	0.78(0.53-1.15)	0.87(0.59-1.27)	0.82(0.55-1.22)	0.73(0.46-1.14)		1.00	0.84(0.56-1.27)	0.75(0.50-1.14)	0.71(0.46-1.08)	0.77(0.51-1.17)	
	≥6	1.00	0.96(0.68-1.34)	1.03(0.74-1.42)	0.95(0.69-1.32)	1.03(0.74-1.45)		1.00	0.99(0.73-1.32)	1.12(0.83-1.50)	0.90(0.65-1.23)	0.93(0.66-1.29)	

Table S6C. Adjusted hazard ratios (HR) in DASH pattern and Alcohol-wheat products pattern in the subgroup analysis[2].

Subgroup		DASH pattern					*P* for interaction	Alcohol-wheat products pattern					*P* for interaction
		**Q1**	**Q2**	**Q3**	**Q4**	**Q5**		**Q1**	**Q2**	**Q3**	**Q4**	**Q5**	
Sex	Female	1.00	**0.50(0.34-0.71)**	**0.62(0.44-0.86)**	**0.48(0.33-0.68)**	**0.37(0.26-0.53)**	**0.001**	1.00	0.94(0.68-1.31)	1.10(0.84-1.61)	**1.64(1.20-2.24)**	**1.53(1.04-2.26)**	0.813
	Male	1.00	1.19(0.90-1.58)	1.10(0.82-1.50)	0.96(0.71-1.31)	0.97(0.70-1.34)		1.00	1.03(0.71-1.48)	1.14(0.81-1.60)	1.36(1.00-1.87)	1.18(0.87-1.62)	
Age	younger	1.00	0.93(0.71-1.22)	0.98(0.75-1.26)	0.75(0.57-1.00)	**0.68(0.51-0.92)**	0.416	1.00	1.02(0.76-1.37)	**1.40(1.05-1.85)**	**1.67(1.28-2.19)**	**1.51(1.13-2.02)**	0.341
	elder	1.00	0.85(0.58-1.25)	0.81(0.55-1.19)	0.92(0.62-1.38)	0.76(0.50-1.15)		1.00	0.88(0.58-1.35)	0.86(0.57-1.32)	1.14(0.77-1.68)	1.04(0.70-1.55)	
BMI (kg/m²)	<24	1.00	0.73(0.48-1.12)	0.90(0.60-1.35)	0.66(0.42-1.03)	**0.55(0.35-0.88)**	0.756	1.00	1.11(0.78-1.57)	1.06(0.75-1.51)	1.36(0.97-1.91)	1.25(0.87-1.80)	0.393
	≥ 24	1.00	0.98(0.75-1.27)	0.92(0.72-1.19)	0.84(0.64-1.11)	**0.74(0.56-0.99)**		1.00	0.87(0.62-1.21)	1.21(0.89-1.65)	**1.56(1.16-2.08)**	1.32(0.97-1.80)	
Region	Urban	1.00	0.94(0.57-1.54)	1.18(0.75-1.85)	1.02(0.65-1.61)	0.74(0.46-1.19)	0.156	1.00	1.01(0.67-1.52)	1.32(0.89-1.95)	1.30(0.86-1.94)	1.37(0.90-2.09)	0.434
	Rural	1.00	0.87(0.67-1.12)	0.80(0.63-1.03)	**0.66(0.49-0.87)**	**0.67(0.50-0.90)**		1.00	0.99(0.73-1.34)	1.09(0.81-1.45)	**1.60(1.22-2.08)**	1.30(0.98-1.72)	
Family history of diabetes	No	1.00	0.89(0.70-1.13)	0.90(0.71-1.13)	0.86(0.68-1.09)	**0.73(0.56-0.94)**	0.056	1.00	1.02(0.80-1.32)	1.17(0.91-1.50)	**1.50(1.19-1.89)**	**1.36(1.06-1.74)**	0.698
	Yes	1.00	1.05(0.54-2.06)	1.18(0.61-2.29)	0.35(0.15-0.83)	0.59(0.28-1.25)		1.00	0.66(0.29-1.51)	1.16(0.57-2.34)	1.61(0.82-3.19)	1.17(0.57-2.40)	

Current smokers	No	1.00	0.83(0.63-1.10)	0.81(0.62-1.05)	**0.71(0.53-0.94)**	**0.58(0.44-0.78)**	0.131	1.00	1.01(0.77-1.32)	1.22(0.94-1.60)	**1.56(1.20-2.02)**	1.35(1.00-1.83)	0.833
	Yes	1.00	1.02(0.70-1.50)	1.20(0.84-1.72)	0.93(0.62-1.39)	1.02(0.67-1.55)		1.00	0.85(0.50-1.44)	0.93(0.57-1.52)	1.30(0.85-1.99)	1.12(0.74-1.69)	
Current alcohol drinkers	No	1.00	0.61(0.44-0.85)	0.72(0.53-0.98)	**0.64(0.46-0.90)**	**0.44(0.31-0.64)**	**0.021**	1.00	0.97(0.69-1.36)	1.32(0.96-1.82)	**1.69(1.24-2.31)**	**1.52(1.05-2.21)**	0.677
	Yes	1.00	1.27(0.92-1.74)	1.17(0.86-1.58)	0.97(0.70-1.34)	0.99(0.71-1.39)		1.00	0.95(0.67-1.35)	0.99(0.70-1.38)	1.31(0.96-1.79)	1.12(0.82-1.54)	
Spicy food(days/week)	<1	1.00	0.64(0.41-1.00)	0.93(0.61-1.43)	0.82(0.51-1.32)	0.73(0.46-1.18)	0.285	1.00	0.95(0.62-1.46)	1.03(0.67-1.57)	1.08(0.70-1.66)	0.76(0.46-1.26)	0.223
	1-5y	1.00	0.89(0.55-1.44)	0.79(0.51-1.24)	**0.60(0.37-0.96)**	**0.52(0.32-0.85)**		1.00	1.12(0.72-1.76)	1.17(0.75-1.82)	**1.57(1.03-2.40)**	**1.90(1.21-2.98)**	
	≥6	1.00	1.03(0.76-1.41)	0.97(0.72-1.32)	0.84(0.60-1.17)	**0.74(0.51-1.06)**		1.00	0.95(0.64-1.41)	1.36(0.94-1.96)	**1.77(1.27-2.48)**	**1.44(1.01-2.04)**	
serum creatinine	normal	1.00	0.92(0.73-1.16)	0.95(0.76-1.18)	0.85(0.67-1.08)	**0.77(0.60-0.98)**	0.110	1.00	1.03(0.80-1.32)	1.25(0.98-1.59)	**1.55(1.23-1.95)**	**1.39(1.09-1.78)**	0.124
	abnormal	1.00	0.77(0.32-1.85)	0.76(0.34-1.72)	**0.24(0.08-0.69)**	**0.14(0.04-0.51)**		1.00	0.50(0.20-1.26)	0.39(0.15-1.02)	0.94(0.45-1.99)	0.50(0.17-1.52)	
systolic blood pressure	normal	1.00	0.85(0.64-1.13)	0.89(0.68-1.17)	0.75(0.56-1.01)	**0.65(0.48-0.88)**	0.936	1.00	1.01(0.76-1.34)	1.23(0.93-1.63)	1.23(0.93-1.62)	**1.38(1.03-1.84)**	**0.004**
	abnormal	1.00	0.99(0.70-1.43)	0.97(0.68-1.38)	0.79(0.53-1.17)	0.80(0.53-1.19)		1.00	0.91(0.58-1.43)	1.11(0.73-1.70)	**2.09(1.44-3.05)**	1.22(0.81-1.84)	
diastolic blood pressure	normal	1.00	0.84(0.66-1.08)	0.86(0.68-1.10)	0.78(0.61-1.01)	**0.68(0.52-0.89)**	0.357	1.00	0.98(0.75-1.27)	1.10(0.85-1.42)	**1.42(1.12-1.81)**	1.30(1.00-1.68)	0.619
	abnormal	1.00	1.21(0.73-2.00)	1.25(0.75-2.09)	0.81(0.46-1.43)	0.70(0.38-1.28)		1.00	0.92(0.47-1.79)	1.60(0.87-2.92)	1.80(1.01-3.21)	1.51(0.84-2.72)	
total cholesterol	normal	1.00	0.87(0.68-1.11)	0.85(0.67-1.08)	**0.76(0.59-0.98)**	**0.73(0.56-0.94)**	0.148	1.00	1.01(0.77-1.31)	1.15(0.89-1.49)	**1.51(1.19-1.92)**	**1.36(1.05-1.75)**	0.922
	abnormal	1.00	1.04(0.60-1.81)	1.22(0.73-2.03)	0.78(0.43-1.44)	**0.51(0.27-0.98)**		1.00	0.88(0.47-1.64)	1.25(0.71-2.21)	1.48(0.85-2.58)	1.24(0.69-2.22)	
triglyceride	normal	1.00	0.96(0.74-1.24)	0.91(0.7-1.17)	0.87(0.66-1.14)	0.76(0.57-1.01)	0.552	1.00	0.94(0.71-1.26)	1.18(0.90-1.54)	**1.43(1.11-1.85)**	**1.51(1.15-1.99)**	0.064
	abnormal	1.00	0.74(0.47-1.15)	0.92(0.62-1.37)	**0.61(0.38-0.96)**	**0.57(0.36-0.92)**		1.00	0.99(0.63-1.55)	1.09(0.68-1.74)	**1.57(1.03-2.41)**	0.92(0.58-1.46)	
low density	normal	1.00	0.89(0.71-1.12)	0.85(0.68-1.07)	0.79(0.62-1.00)	**0.70(0.54-0.90)**	0.059	1.00	0.99(0.77-1.27)	1.12(0.87-1.43)	1.50(1.20-1.89)	**1.36(1.06-1.73)**	0.095

lipoprotein cholesterol	abnormal	1.00	0.93(0.4-2.15)	1.78(0.83-3.80)	0.69(0.27-1.74)	0.54(0.21-1.39)		1.00	1.02(0.44-2.36)	1.70(0.79-3.65)	1.45(0.65-3.22)	0.97(0.41-2.29)	
high density lipoprotein cholesterol	normal	1.00	0.95(0.75-1.21)	1.02(0.81-1.29)	0.87(0.67-1.11)	**0.76(0.59-0.99)**	0.253	1.00	0.95(0.73-1.23)	1.15(0.90-1.48)	**1.43(1.13-1.82)**	**1.39(1.08-1.79)**	0.471
	abnormal	1.00	0.85(0.48-1.51)	0.63(0.37-1.09)	0.56(0.30-1.05)	0.65(0.35-1.22)		1.00	0.94(0.50-1.78)	1.12(0.59-2.12)	1.64(0.93-2.88)	0.87(0.46-1.66)	

Table S6D. Adjusted hazard ratios (HR) in Meat pattern and Dairy products-eggs pattern in the subgroup analysis[2].

Subgroup		Meat pattern					*P* for interaction	Dairy products-eggs pattern					*P* for interaction
		Q1	Q2	Q3	Q4	Q5		Q1	Q2	Q3	Q4	Q5	
Sex	Female	1.00	0.84(0.63-1.12)	0.80(0.59-1.09)	0.76(0.54-1.06)	0.80(0.54-1.18)	0.535	1.00	0.76(0.54-1.07)	0.78(0.55-1.09)	0.72(0.51-1.02)	**0.58(0.40-0.83)**	0.136
	Male	1.00	1.07(0.76-1.51)	1.07(0.78-1.48)	1.13(0.82-1.55)	1.22(0.88-1.7)		1.00	1.01(0.77-1.31)	1.04(0.79-1.37)	1.06(0.80-1.42)	1.14(0.84-1.54)	
Age	younger	1.00	0.87(0.66-1.15)	0.88(0.67-1.16)	0.98(0.74-1.28)	1.00(0.74-1.35)	0.653	1.00	0.89(0.69-1.15)	0.89(0.68-1.15)	0.81(0.61-1.06)	0.85(0.64-1.13)	0.153
	elder	1.00	1.00(0.70-1.44)	1.04(0.72-1.49)	0.84(0.57-1.25)	1.10(0.73-1.67)		1.00	1.02(0.69-1.51)	1.09(0.74-1.60)	1.33(0.91-1.95)	0.97(0.64-1.46)	
BMI (kg/m²)	<24	1.00	0.96(0.65-1.42)	1.13(0.76-1.67)	1.04(0.69-1.57)	1.35(0.86-2.13)	0.916	1.00	0.96(0.64-1.43)	0.89(0.59-1.36)	0.97(0.63-1.48)	0.83(0.53-1.28)	0.978
	≥ 24	1.00	0.94(0.72-1.23)	0.89(0.68-1.16)	0.93(0.72-1.22)	0.93(0.7-1.24)		1.00	0.96(0.75-1.24)	0.98(0.76-1.25)	0.96(0.74-1.24)	0.92(0.70-1.21)	
Region	Urban	1.00	1.04(0.73-1.47)	1.07(0.75-1.52)	0.79(0.53-1.19)	1.03(0.66-1.62)	0.263	1.00	1.22(0.76-1.95)	1.30(0.83-2.05)	1.17(0.74-1.85)	1.32(0.84-2.08)	0.292
	Rural	1.00	0.87(0.65-1.14)	0.87(0.66-1.14)	1.00(0.77-1.31)	0.99(0.74-1.32)		1.00	0.90(0.71-1.15)	0.84(0.66-1.09)	0.92(0.71-1.19)	**0.74(0.55-0.99)**	
Family history of diabetes	No	1.00	0.90(0.71-1.13)	0.91(0.72-1.14)	0.92(0.73-1.17)	1.03(0.80-1.33)	0.838	1.00	0.91(0.73-1.14)	0.93(0.74-1.17)	0.95(0.75-1.21)	0.98(0.77-1.25)	0.061
	Yes	1.00	1.14(0.56-2.31)	1.21(0.62-2.37)	1.18(0.60-2.32)	1.06(0.49-2.28)		1.00	1.33(0.71-2.50)	0.97(0.49-1.92)	0.88(0.45-1.74)	0.48(0.22-1.02)	

Current smokers	No	1.00	0.86(0.67-1.1)	0.94(0.73-1.20)	0.74(0.56-0.98)	0.94(0.69-1.28)	0.002	1.00	0.96(0.74-1.26)	0.88(0.67-1.16)	0.94(0.71-1.24)	0.84(0.63-1.12)	0.428
	Yes	1.00	1.40(0.85-2.31)	1.12(0.69-1.81)	1.66(1.06-2.58)	1.35(0.85-2.13)		1.00	0.89(0.63-1.27)	1.09(0.77-1.54)	0.93(0.64-1.36)	1.08(0.73-1.60)	
Current alcohol drinkers	No	1.00	0.87(0.65-1.16)	0.86(0.63-1.16)	0.82(0.59-1.14)	0.89(0.61-1.30)	0.344	1.00	0.97(0.71-1.33)	0.98(0.72-1.36)	0.82(0.58-1.15)	0.83(0.59-1.18)	0.465
	Yes	1.00	1.05(0.75-1.46)	1.04(0.75-1.44)	1.10(0.80-1.52)	1.13(0.81-1.58)		1.00	0.92(0.69-1.23)	0.92(0.69-1.22)	1.08(0.81-1.44)	0.96(0.70-1.30)	
Spicy food(days/week)	<1	1.00	1.07(0.72-1.60)	0.92(0.58-1.45)	0.90(0.56-1.45)	1.64(1.00-2.7)	0.603	1.00	0.90(0.58-1.38)	0.98(0.63-1.54)	1.01(0.65-1.56)	0.89(0.55-1.42)	0.923
	1-5y	1.00	0.72(0.48-1.08)	0.88(0.60-1.30)	0.83(0.55-1.26)	0.70(0.44-1.12)		1.00	0.91(0.60-1.39)	0.75(0.49-1.15)	0.76(0.49-1.17)	0.76(0.49-1.17)	
	≥6	1.00	0.96(0.68-1.35)	0.94(0.67-1.31)	0.97(0.70-1.35)	0.97(0.68-1.37)		1.00	1.01(0.75-1.36)	1.06(0.79-1.44)	1.02(0.74-1.41)	0.94(0.67-1.33)	
serum creatinine	normal	1.00	0.94(0.75-1.18)	0.98(0.78-1.23)	1.00(0.80-1.26)	1.08(0.84-1.39)	0.038	1.00	0.95(0.77-1.19)	0.96(0.77-1.20)	1.02(0.81-1.27)	0.94(0.74-1.19)	0.246
	abnormal	1.00	0.74(0.35-1.57)	0.48(0.19-1.17)	0.32(0.10-1.01)	0.39(0.12-1.27)		1.00	0.80(0.35-1.83)	0.66(0.27-1.59)	0.23(0.07-0.77)	0.50(0.19-1.33)	
systolic blood pressure	normal	1.00	1.02(0.78-1.34)	0.94(0.71-1.23)	1.04(0.78-1.37)	1.17(0.87-1.59)	0.564	1.00	0.85(0.65-1.10)	0.83(0.63-1.09)	0.84(0.64-1.10)	0.81(0.61-1.08)	0.682
	abnormal	1.00	0.75(0.51-1.1)	0.95(0.67-1.35)	0.83(0.57-1.20)	0.83(0.56-1.24)		1.00	1.10(0.77-1.57)	1.19(0.84-1.70)	1.07(0.72-1.57)	1.14(0.77-1.67)	
diastolic blood pressure	normal	1.00	0.92(0.73-1.17)	0.86(0.67-1.09)	0.85(0.67-1.09)	1.04(0.8-1.35)	0.110	1.00	0.97(0.76-1.22)	0.91(0.71-1.16)	0.95(0.75-1.22)	0.94(0.73-1.21)	0.555
	abnormal	1.00	0.99(0.55-1.80)	1.46(0.85-2.51)	1.34(0.78-2.29)	0.89(0.48-1.66)		1.00	0.90(0.56-1.47)	1.20(0.74-1.93)	0.92(0.54-1.56)	0.74(0.41-1.34)	
total cholesterol	normal	1.00	1.01(0.80-1.28)	1.01(0.80-1.28)	0.98(0.77-1.25)	1.03(0.79-1.35)	0.278	1.00	0.97(0.77-1.22)	0.98(0.78-1.24)	0.98(0.77-1.24)	0.97(0.76-1.25)	0.715
	abnormal	1.00	0.56(0.31-1.01)	0.60(0.35-1.03)	0.72(0.42-1.25)	0.95(0.54-1.67)		1.00	0.88(0.52-1.48)	0.87(0.50-1.50)	0.88(0.51-1.53)	0.60(0.33-1.08)	
triglyceride	normal	1.00	0.85(0.66-1.10)	0.98(0.76-1.25)	0.89(0.68-1.15)	0.96(0.72-1.27)	0.264	1.00	1.01(0.79-1.30)	1.02(0.79-1.32)	1.00(0.77-1.30)	0.96(0.73-1.26)	0.836
	abnormal	1.00	1.20(0.78-1.86)	0.81(0.52-1.27)	1.04(0.67-1.63)	1.17(0.72-1.89)		1.00	0.83(0.56-1.23)	0.80(0.54-1.19)	0.88(0.58-1.34)	0.79(0.5-1.23)	
low density	normal	1.00	0.96(0.77-1.21)	0.94(0.75-1.18)	0.97(0.77-1.22)	1.04(0.81-1.34)	0.703	1.00	0.95(0.76-1.19)	0.96(0.76-1.20)	0.98(0.78-1.24)	0.94(0.74-1.2)	0.944

lipoprotein cholesterol	abnormal	1.00	0.59(0.26-1.36)	0.65(0.30-1.39)	0.47(0.20-1.08)	0.64(0.28-1.45)		1.00	0.85(0.40-1.81)	1.03(0.48-2.22)	0.62(0.27-1.41)	0.42(0.17-0.99)	
high density lipoprotein cholesterol	normal	1.00	0.92(0.73-1.16)	0.97(0.77-1.22)	0.91(0.72-1.16)	1.01(0.78-1.31)	0.471	1.00	1.03(0.82-1.30)	0.97(0.76-1.23)	1.04(0.82-1.32)	0.91(0.71-1.18)	0.133
	abnormal	1.00	1.2(0.67-2.17)	0.65(0.34-1.24)	1.08(0.60-1.97)	1.00(0.53-1.9)		1.00	0.55(0.31-0.97)	0.80(0.48-1.33)	0.55(0.29-1.06)	0.93(0.53-1.65)	

[1] Adjusted hazard ratios (HR) and 95% confidence intervals (CI) of total DP scores obtained using Cox proportional hazard regression. Final model was adjusted for sex, age (<60 or≥60years old), region (urban or rural), educational level (primary school or below, Middle school or high school, high school above), household annual income (<20000, 20000-99999, 10000-19999,≥20000 CNY/year), family history of diabetes, BMI (standard and below, overweight and above) weekly physical activity (<10.0,≥10.0 METs-h per week), smoking, drinking alcohol, drinking tea, eating spicy food (<1, 1-5,≥6 days per week), and daily total energy intake (kcal/day).

[2] Adjusted hazard ratios (HR) and 95% confidence intervals (CI) of total DP scores obtained using Cox proportional hazard regression. Final model was adjusted for sex, age (<60 or≥60years old), region (urban or rural), educational level (primary school or below, Middle school or high school, high school above), household annual income (<20000, 20000-99999, 10000-19999,≥20000 CNY/year), family history of diabetes, BMI (standard and below, overweight and above) weekly physical activity (<10.0,≥10.0 METs-h per week), smoking, drinking alcohol, drinking tea, eating spicy food (<1, 1-5,≥6 days per week), daily total energy intake (kcal/day),serum creatinine (Cr)(male in normal:20-59years old is 57-97μmol/L, 60-79 years old is 57-111μmol/L; female in normal:20-59years old is 41-73μmol/L, 60-79 years old is 41-81μmol/L, beyond this range is abnormal), systolic blood pressure (SBP)(90-140mmHg for normal, beyond this range is abnormal), diastolic blood pressure (DBP)(60-90mmHg for normal, beyond this range is abnormal),triglyceride (TG)(0.56~1.70 mmol/L for normal, beyond this range is abnormal),total cholesterol (TC)(2.84~5.68 mmol/L for normal, beyond this range is abnormal), low density lipoprotein cholesterol (LDL-C)(2.10~3.10 mmol/L for normal, beyond this range is abnormal) and high density lipoprotein cholesterol (HDL-C)(1.14~1.76 mmol/L for normal, beyond this range is abnormal).
